# Nereididae (Annelida) of the Coral Triangle, Indonesia, with some notes on nyale worms, new genus records, and a review of national species discovery

**DOI:** 10.3897/zookeys.1296.201110

**Published:** 2026-11-06

**Authors:** Joko Pamungkas, Christopher J. Glasby, Geterudis Kerans, Ludi P. Aji, Ekin Tilic

**Affiliations:** 1 Directorate of Scientific Collection Management, National Research and Innovation Agency, Jakarta, Indonesia Directorate of Scientific Collection Management, National Research and Innovation Agency Jakarta Indonesia; 2 Senckenberg Research Institute and Natural History Museum, Frankfurt, Germany Senckenberg Research Institute and Natural History Museum Frankfurt Germany; 3 Museum and Art Gallery of the Northern Territory, Darwin, Australia Museum and Art Gallery of the Northern Territory Darwin Australia; 4 Australian Museum, Sydney, Australia Australian Museum Sydney Australia; 5 Catholic University of Weetebula, East Nusa Tenggara, Indonesia Catholic University of Weetebula East Nusa Tenggara Indonesia; 6 Research Center for Ecology, National Research and Innovation Agency, Jakarta, Indonesia Research Center for Ecology, National Research and Innovation Agency Jakarta Indonesia

**Keywords:** Identification key, marine biodiversity, new records, Polychaeta, taxonomy, *wawo*

## Abstract

Taxonomic information on Indonesian polychaetes is limited. In the present work, 20 nereidid species (10 genera) from ten locations within the Indonesian part of the Coral Triangle were identified. The specimens were collected between 2009 and 2018 from intertidal reef (mostly) and mangrove habitats. The genera with the most species were *Perinereis* (6 species), followed by *Neanthes* (5 species), and the most common species were *Perinereis helleri* (Grube, 1878), *Perinereis larentukana* (Grube, 1881), *Perinereis obfuscata* (Grube, 1878) and *Pseudonereis trimaculata* (Horst, 1924). Two genera represent first records for Indonesian waters: *Cheilonereis* sp. and *Hediste* sp., and three species are potentially new to science: *Cheilonereis* sp., *Hediste* sp., and Neanthes cf. gisserana. Five *nyale* species (reproductive polychaete swarms in Nusa Tenggara) were identified: *Composetia marmorata* (Horst, 1924), Neanthes cf. gisserana, *Neanthes unifasciata* (Willey, 1905), *P. helleri*, and *P. larentukana*. These findings suggest that *nyale* are mostly the same species as *wawo* (reproductive polychaete swarms in Maluku) and hence refute the common belief in central and eastern Indonesia that both groups of worms are unique to Nusa Tenggara and Maluku, respectively. During the period of circa 1.5 centuries (1865–2025), 84 Indonesian nereidid species (24 genera in 29 publications) have been identified, 44 of which (15 genera in 16 publications) are new to science. Despite its status as a country with mega-marine biodiversity, Indonesia’s contribution to global nereidid species discovery has been disproportionately small. To expedite the discovery of additional Indonesian nereidid species potentially new to science, further studies involving integrative taxonomic studies should be concentrated on the three big islands with the least data, i.e., Sumatra, Kalimantan, and Papua. The provided key to all genera of Nereididae in Indonesian waters will facilitate preliminary identification.

## Introduction

Nereididae Blainville, 1818, is the third-most species-rich polychaete family after Syllidae Grube, 1850, and Polynoidae Kinberg, 1856 (Pamungkas et al. 2019), with ~ 720 species in ~ 50 genera described worldwide (WoRMS Editorial Board 2026). Nereidid species are known to occupy a great range of habitats from shallow estuarine and marine environments to the deep-sea. Members of the subfamily Namanereidinae, in particular, also inhabit a diverse variety of freshwater habitats (Glasby et al. 2009).

In contrast to the global diversity of the family, the number of nereidid species recorded from Indonesia is quite limited, i.e., only 75 species (Pamungkas and Glasby 2019), despite the fact that the country is part of the Coral Triangle, a marine region recognised as the global hotspot of marine biodiversity (e.g., Roberts et al. 2002; Hoeksema 2007; Asaad et al. 2020). The Coral Triangle itself is part of the Indo-Malay-Philippines Archipelago (IMPA), which encompasses mainland Southeast Asia, extending almost to Australia, including their marine areas (Glasby and Al-Hakim 2017). More taxonomic efforts are hence required to reveal the biodiversity of Indonesian nereidids.

Adult nereidids generally live as benthic animals. However, certain species undergo metamorphosis as they mature (becoming heteronereids) and then swim to surface waters, triggering mass spawning events that are often synchronized with lunar cycles (e.g., Fong 1993; Hardege and Bartels-Hardege 1995; although moon phase seems to be the initial ‘trigger’, it may be that some polychaetes are also responding to other polychaetes spawning). With some other polychaete families (especially Eunicidae), nereidids that spawn en masse are known as ‘*wawo*’ in Maluku Province, or ‘*nyale*’ in East and West Nusa Tenggara Provinces – the worms are considered a special delicacy by the locals as the animals only occur annually. While the taxonomy of nereidid *wawo* has been investigated (e.g., Martens et al. 1995; Pamungkas 2015; Pamungkas and Glasby 2015), *nyale* is poorly studied. Some local researchers identify *nyale* only as eunicids (e.g., Bachtiar and Bachtiar 2019; Bachtiar and Odani 2021). However, the identification needs to be verified as the focus of these studies was on the prediction of the occurrence of *nyale*.

In the present work, we identified nereidid species collected from the Indonesian parts of the Coral Triangle, including *nyale* for the first time. Additionally, we reviewed the Indonesian nereidid species discovery rate based on previously published taxonomic literature to assess its progress over the decades, and mapped their geographic distribution to determine which regions have the most and least data.

## Materials and methods

### Study area and sample collection

**Figure 1.**
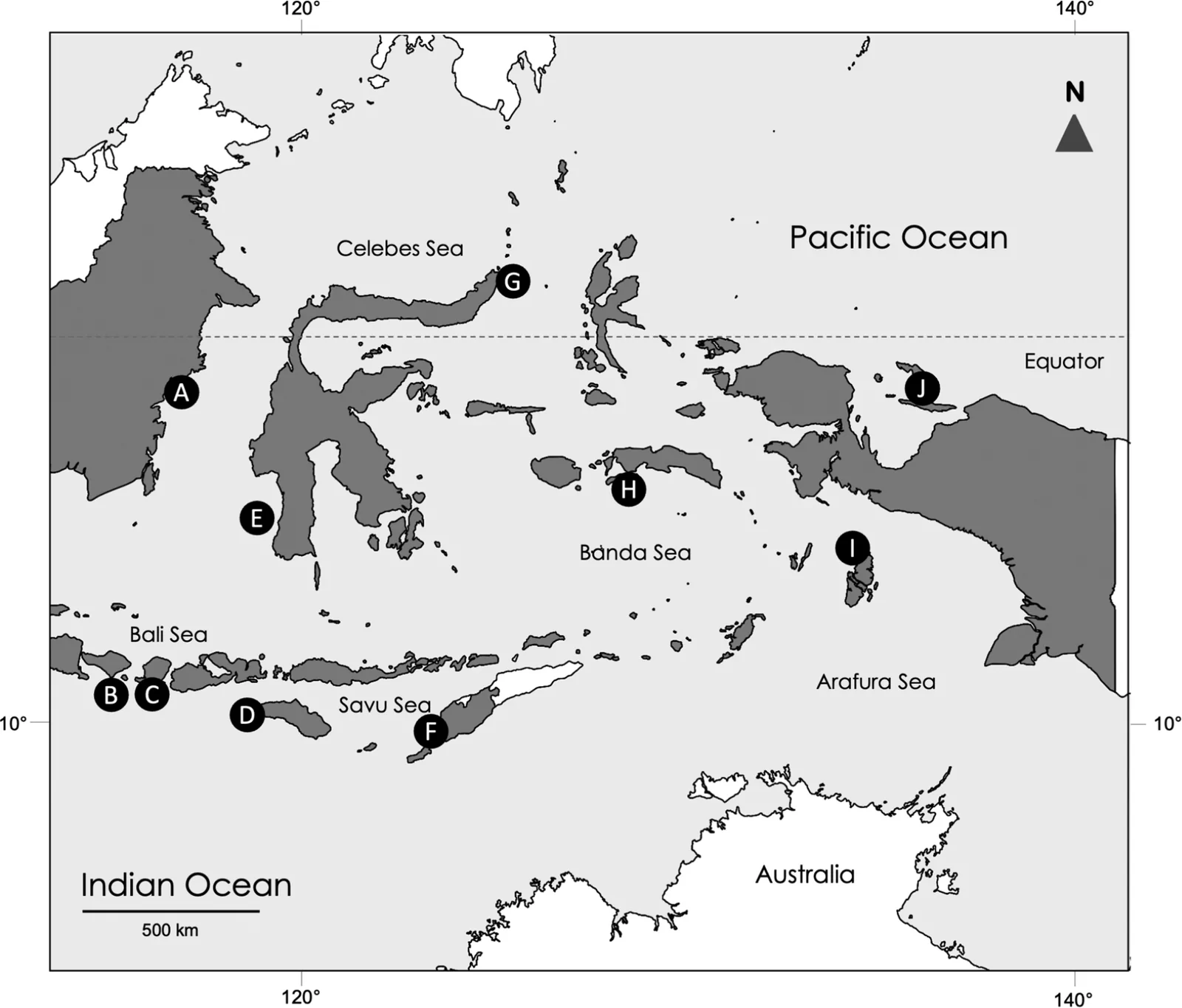
Sampling sites. **A**. Balikpapan (East Kalimantan); **B**. Badung (Bali); **C**. Central Lombok (West Nusa Tenggara); **D**. Southwest Sumba (East Nusa Tenggara); **E**. Makassar (South Sulawesi); **F**. Kupang (East Nusa Tenggara); **G**. Bitung (North Sulawesi); **H**. Ambon and Central Maluku (Maluku); **I**. Aru Islands (Maluku); **J**. Biak (Papua).

The specimens obtained in the present study were collected from 10 locations in the Indonesian parts of the Coral Triangle between 2009 and 2018 (Fig. 1). Polychaete specimens were collected from intertidal reef habitats during low tide. To obtain the specimens, reef rubble was broken using a hammer and a chisel. The worms were placed in jars with 4% formalin. Smaller pieces of rubble, which usually contain smaller worms, were placed in a bucket with seawater and left for ~ 2 h for deoxygenation. The water was stirred vigorously, then poured onto a sieve with 0.5 mm mesh size. This step was repeated three times. Material retained on the sieve was gently moved into a jar filled with 4% formalin using a plastic spoon. In addition, *nyale* worms, i.e., heteronereids consumed by the natives of Lombok and Sumba Islands, were collected using a fishing net. The worms were then placed directly into a jar containing 4% formalin. After 24 h, all the specimens soaked in formalin were rinsed in tap water and preserved in 70% ethanol.

### Species identification and deposition

Preserved specimens were examined using both a Leica WILD MZ8 stereo microscope and a Leica Leitz DIAPLAN compound microscope. Photomicrographs were taken with a Canon EOS M6 DSLR camera mounted on both microscopes. Photo stacking was performed online at focusstackingonline.com. Initial identification to the genus level was made using the key of Wilson et al. (2023). In this study, the diagnoses separate the given operational taxonomic unit from other known Indonesian Nereididae rather than from other species known globally in that genus. The specimens were deposited at the Museum Zoologicum Bogoriense (**MZB**) in Cibinong, Bogor, West Java, Indonesia, and the Naturmuseum Senckenberg (**SMF**) in Frankfurt am Main, Germany.

### Compilation of Indonesian nereidid species records

We garnered data on Indonesian nereidid species published in taxonomic literature, with some other information (e.g., locality/sampling locations, expedition/collector, repository, habitat). In the present study, only nereidids identified to the species level were included. Species identified to morphospecies (e.g., *Nereis* sp.) were not included, unless they represent the only current record of the genus, in which case their inclusion was considered useful without unduly inflating species numbers. New genus records discovered in the present study were therefore also added to the data. The validity of species names was verified on the World Register of Marine Species (WoRMS) at www.marinespecies.org using the ‘Match taxa’ feature. Records of Indonesian nereidid species were mapped using SimpleMappr at www.simplemappr.net based on the coordinates provided (if they are not provided, approximate locations were estimated). Acronyms of institutional repositories housing Indonesian nereidid collections are provided in Table 1.

**Table 1.** Acronym of institutional repositories housing Indonesian nereidid collections.

**Acronym**	**Institution**	**City**	**Country**
NHMUK	Natural History Museum (formerly British Museum of Natural History)	London	England
LKCNHM	Lee Kong Chian Natural History Museum	Singapore	Singapore
MZB*	Museum Zoologicum Bogoriense	Bogor	Indonesia
NBC	Naturalis Biodiversity Center	Leiden	Netherlands
NRS	Naturhistoriska Riksmuseet	Stockholm	Sweden
NTM	Northern Territory Museum	Darwin	Australia
RCLA**	Reference Collection LIPI Ambon	Ambon	Indonesia
SMF	Senckenberg Forschungsinstitut und Naturmuseum	Frankfurt am Main	Germany
USNM	United States National Museum	Washington, D.C.	USA
ZMB	Museum für Naturkunde	Berlin	Germany
ZMH	Zoological Museum Hamburg	Hamburg	Germany
ZMUC	Zoological Museum, University of Copenhagen	Copenhagen	Denmark
ZRC	Zoological Reference Collection	Singapore	Singapore

* This institution is now under the Directorate of Scientific Collection Management, National Research and Innovation Agency. ** All marine collections at this institution are now being moved to MZB.

## Results

### Nereidid species richness

We identified 20 nereidid species (10 genera) from specimens collected in the Coral Triangle. The genera with the most species were *Perinereis* (6 species), followed by *Neanthes* (5 species). The most common species were *Perinereis helleri* (Grube, 1878) and *Pseudonereis trimaculata* (Horst, 1924), which occurred at five and four sampling sites, respectively, followed by *Composetia marmorata* (Horst, 1924), *Perinereis larentukana* (Grube, 1881), *Perinereis nigropunctata* (Horst, 1889) and *Perinereis obfuscata* (Grube, 1878), which occurred at three sampling sites (Table 2). *Cheilonereis* sp. and *Hediste* sp. were first records from Indonesia. Three species are potentially new to science: *Cheilonereis* sp., *Hediste* sp., and Neanthes cf. gisserana. In addition, Neanthes cf. masalacensis, a *wawo* species identified by Pamungkas and Glasby (2015), is also potentially new to science.

**Table 2.** Nereidid species recorded at each sampling site. Species in bold are potentially new to science. Sampling sites as shown in Fig. 1.

**No**.	**Species**	**Sampling sites**									
		**A**	**B**	**C**	**D**	**E**	**F**	**G**	**H**	**I**	**J**
1	*Alitta* sp.	-	-	-	-	-	-	-	-	+	-
2	*Ceratonereis* sp. 1	-	-	-	-	-	-	-	+	+	-
3	*Ceratonereis* sp. 2	-	-	-	-	+	-	-	-	-	-
4	***Cheilonereis* sp**.*	-	-	-	-	-	-	-	-	+	-
5	*Composetia marmorata* (Horst, 1924)**	-	-	+	-	-	-	-	+	+	-
6	***Hediste* sp**.*	-	-	-	-	-	-	-	-	+	-
7	Neanthes cf. gisserana**	-	-	+	-	-	-	-	-	-	-
8	*Neanthes unifasciata* (Willey, 1905)**	-	-	+	-	-	-	-	-	+	-
9	*Neanthes* sp. 1	-	-	-	-	-	-	-	-	+	-
10	*Neanthes* sp. 2	-	-	-	-	-	-	+	-	-	-
11	*Neanthes* sp. 3	-	-	-	-	-	-	-	+	-	-
12	*Perinereis helleri* (Grube, 1878)**	-	-	-	+	+	-	+	+	+	-
13	*Perinereis larentukana* (Grube, 1881)**	-	-	+	+	-	-	-	+	-	-
14	*Perinereis nigropunctata* (Horst, 1889)	-	-	-	-	+	-	-	+	+	-
15	*Perinereis obfuscata* (Grube, 1878)	-	-	-	-	+	-	-	-	+	+
16	*Perinereis singaporiensis* (Grube, 1878)	-	-	-	-	-	-	+	-	-	-
17	*Perinereis* sp.	-	-	-	-	-	-	-	-	+	-
18	*Platynereis* sp.	-	-	-	-	-	-	+	-	-	-
19	*Pseudonereis trimaculata* (Horst, 1924)	+	+	-	-	-	-	+	+	-	-
20	*Solomononereis marauensis* Gibbs, 1971	-	-	-	-	-	-	-	+	-	-

* New record for Indonesia. ** *Nyale* worms.

Five nereidid *nyale* species were identified in the present study. Four species were recorded from West Nusa Tenggara: *Composetia marmorata* (Horst, 1924), Neanthes cf. gisserana, *Neanthes unifasciata* (Willey, 1905), and *P. larentukana*, whereas one species was from East Nusa Tenggara, i.e., *P. helleri*.

### Taxonomic account

#### Alitta

Taxon classificationAnimaliaPhyllodocidaNereididae

sp.

7A60E71D-9652-53EE-A931-078DF3871272

Fig. 2

##### Material examined.

MZB. Pol. 00298 (1 specimen), collected by Joko Pamungkas on 24 November 2017 from Warmar Island: Wangel Village, Aru Islands District, Aru Islands Regency, Maluku Province (5°45'05.1"S, 134°11'43.2"E).

##### Diagnosis.

Single specimen complete, ~ 27 mm long by nearly 2 mm wide at widest part, widening around mid-body, then gradually tapering posteriorly. Body color in alcohol whitish without pigmentation. Prostomium roughly pentagonal with entire anterior margin. Four roughly circular black eyes present with anterior and posterior eyes arranged in an almost longitudinal row. Antennae conical. Palps biarticulated with massive subconical palpophores having a single transverse groove, and subconical to oval palpostyles. Tentacular belt length greater than that of chaetiger 1. Four pairs of smooth peristomial cirri with distinct cirrophores present; longest cirri reaching chaetiger 2. Paragnaths present on both rings and arranged as follows. Area I = 1; Area II = 8; Area III = 6 in a single row; Area IV = 12; Area V = 0; Area VI = 4–7 in a roughly circular group; Area VII = 5 in a single row; Area VIII = 2 in a single row. Dorsal notopodial ligules markedly broader in posterior chaetigers. Notochaetae without sesquigomph spinigers.

**Figure 2.**
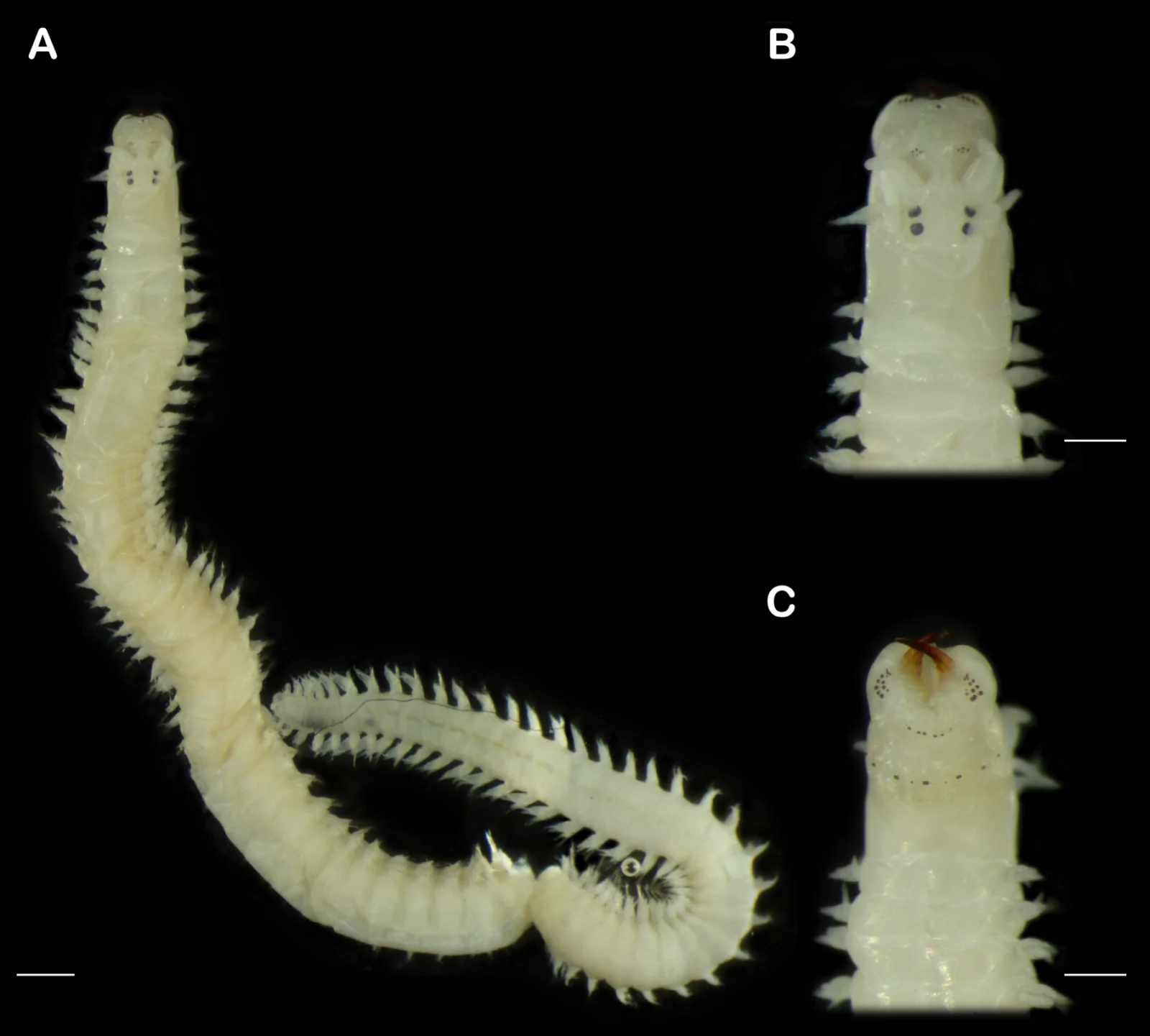
*Allita* sp. **A**. Whole body; **B**. Anterior end with everted proboscis (dorsal view); **C**. Anterior end with everted proboscis (ventral view). Scale bars: 1 mm (**A**); 0.5 mm (**B, C**).

##### Distribution and habitat.

The species was obtained from Maluku, Indonesia, inhabiting intertidal rocky shore.

##### Remarks.

To date, there are nine *Alitta* species described worldwide (WoRMS Editorial Board 2026), and only one of them was reported to occur in Indonesia, i.e., *Alitta succinea* (Leuckart, 1847) – see Pflugfelder (1933) and Pamungkas and Glasby (2019). Our specimen fits the minimal generic diagnosis of Wilson et al. (2023), i.e., ‘Dorsal notopodial ligule markedly broader on posterior chaetigers; palpophore massive subconical, flattened (palpostyle is minute by comparison); ventral peristomial flap absent; notochaetae sesquigomph spinigers absent’. Additional collections to obtain more specimens, as well as further identification, are required to confirm the identity of this species. This is the first *Alitta* in the collection of MZB.

#### Ceratonereis

Taxon classificationAnimaliaPhyllodocidaNereididae

sp. 1

E66F0505-05EE-5FE4-A61E-2768EC985403

Fig. 3A, B

##### Material examined.

MZB. Pol. 00274 (5 specimens), collected by Joko Pamungkas on 23 November 2017 from Warmar Island: Siwalima Village, Aru Islands District, Aru Islands Regency, Maluku Province (5°45'26.9"S, 134°13'23.5"E); MZB. Pol. 00275 (5 specimens), collected by Joko Pamungkas on 21 November 2017 from Ambon Island: Leahari Village, South Leitimur District, Ambon City, Maluku Province (3°42'32.1"S, 128°16'10.2"E).

##### Diagnosis.

Body segments gradually tapering posteriorly. Body color in alcohol cream or whitish with bronze pigmentation in some areas, including prostomium (extending to posterior parts of palpophores) and dorsal area of body segments (as transverse bronze striae resembling wood grain; most intense color from chaetigers 1 to 10, then becoming faint posteriorly). Prostomium with anterior margin deeply indented. Four black eyes present and arranged obliquely with distance between anterior eyes wider than that of posterior ones. Anterior eyes reniform; posterior eyes circular. Antennae subulate and multiarticulated. Palps biarticulated with cylindrical palpophores and pyriform palpostyles. Peristomium length greater than that of chaetiger 1. Four pairs of smooth peristomial cirri with distinct cirrophores present; longest cirri reaching chaetiger 12. Dorsal cirri evenly tapering and as long as parapodia. Paragnaths present on maxillary ring only. Notochaetae without homogomph falcigers; sesquigomph falcigers and sesquigomph spinigers present.

**Figure 3.**
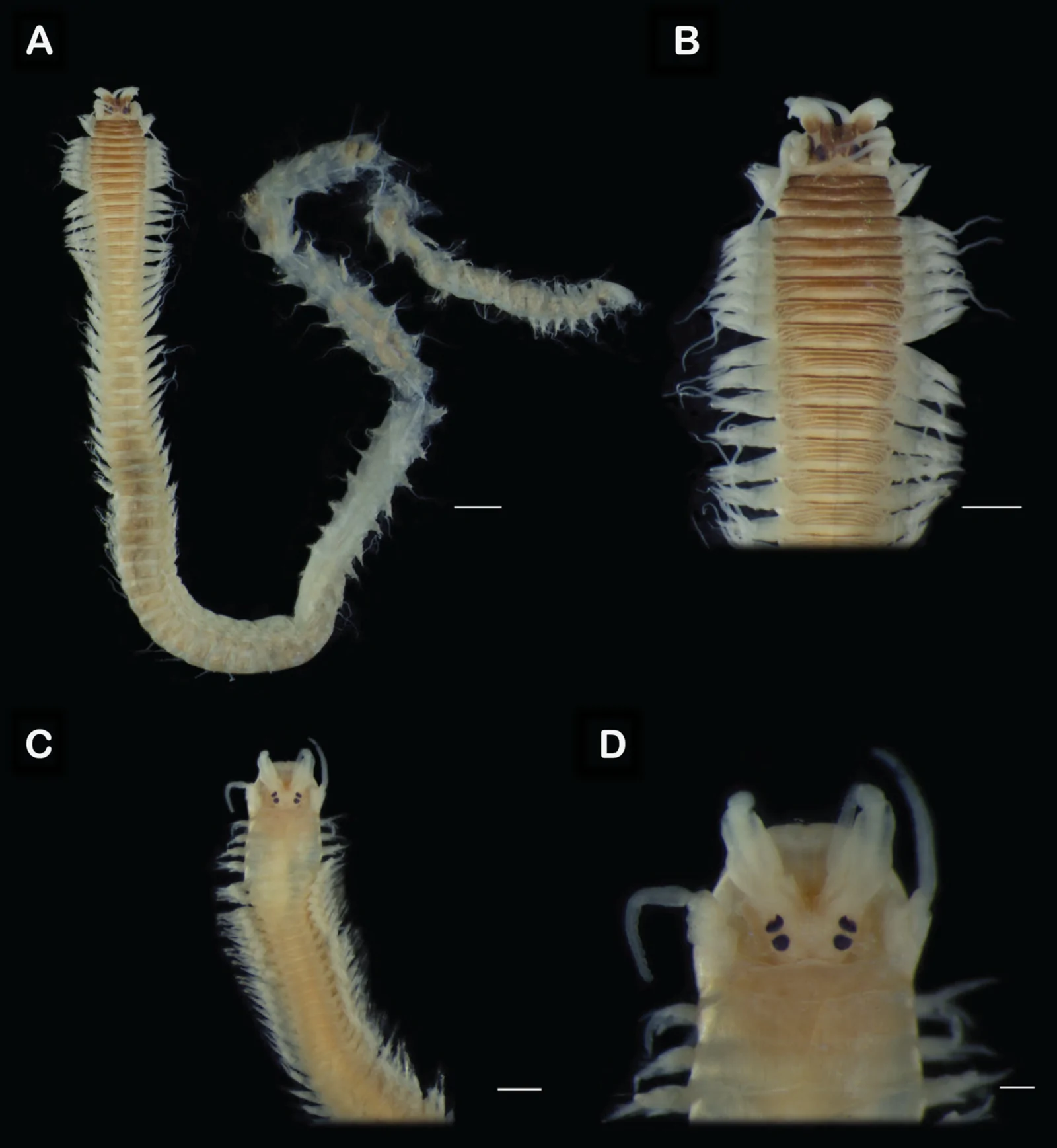
*Ceratonereis* species. **A**. *Ceratonereis* sp. 1 (whole body); **B**. Anterior end (dorsal view); **C**. *Ceratonereis* sp. 2 (dorsal view of anterior end); **D**. Anterior end (dorsal view). Scale bars: 2 mm (**A**); 1 mm (**B, C**); 0.25 mm (**D**).

##### Distribution and habitat.

The species was obtained from Maluku, Indonesia, inhabiting intertidal rocky shore.

##### Remarks.

Five *Ceratonereis* species were previously reported to occur in Indonesia, i.e., *Ceratonereis singularis australis* Hartmann-Schröder, 1985 and Ceratonereis (Ceratonereis) cf. perkinsi Hartmann-Schröder, 1985 among *wawo* worms on Ambon Island – see Martens et al. (1995), Pamungkas (2015), Pamungkas and Glasby (2015). Also, *Ceratonereis dorsolineata* (Horst, 1924), *Ceratonereis ternatensis* (Fischli, 1900), and *Ceratonereis tripartita* Horst, 1918 – all these Indonesian species were described based on their modified reproductive forms, which are suboptimal for accurate species descriptions, in particular because paragnath counts often cannot be made accurately because of pharynx damage and paragnath resorption. Further identification is required to reveal the identity of the present species (comparative material is ideally needed).

#### Ceratonereis

Taxon classificationAnimaliaPhyllodocidaNereididae

sp. 2

A03F8C47-4B55-57A4-979E-BF85D7A32FDF

Fig. 3C, D

##### Material examined.

MZB. Pol. 00318 (1 specimen), collected by Joko Pamungkas on 19 December 2017 from Barrang Lompo Island: Barrang Lompo Village, Sangkarrang Island District, Makassar City, South Sulawesi Province (5°02'45.7"S, 119°19'43.4"E).

##### Diagnosis.

Single specimen incomplete. Body color in alcohol cream, lacking pigmentation pattern of *Ceratonereis* sp. 1, tapering posteriorly to approximately chaetiger 6. Prostomium with anterior margin deeply indented. Four black eyes present, arranged obliquely with distance between anterior eyes wider than that between posterior ones. Anterior eyes egg-shaped and chipped anteriorly; posterior eyes roughly circular. Antennae subulate, as long as palps. Palps biarticulated with cylindrical palpophores and rounded palpostyles. Peristomium length shorter than that of chaetiger 1. Four pairs of articulated peristomial cirri with distinct cirrophores present (most cirri broken). Paragnaths present on maxillary ring only. Dorsal cirri broken. Notochaetae without homogomph falcigers; sesquigomph falcigers and sesquigomph spinigers present.

##### Distribution and habitat.

The species was obtained from South Sulawesi, Indonesia, inhabiting intertidal sandy shore with reefs.

##### Remarks.

With *Ceratonereis* sp. 1, more specimens and comparative material are required to confirm the identity of the present species.

#### Cheilonereis

Taxon classificationAnimaliaPhyllodocidaNereididae

sp.

B33363EE-2E16-58A4-8D9F-849E26228C8A

Fig. 4

##### Material examined.

MZB. Pol. 00294 (2 specimens), collected by Joko Pamungkas on 24 November 2017 from Warmar Island: Wangel Village, Aru Islands District, Aru Islands Regency, Maluku Province (5°45'05.1"S, 134°11'43.2"E).

**Figure 4.**
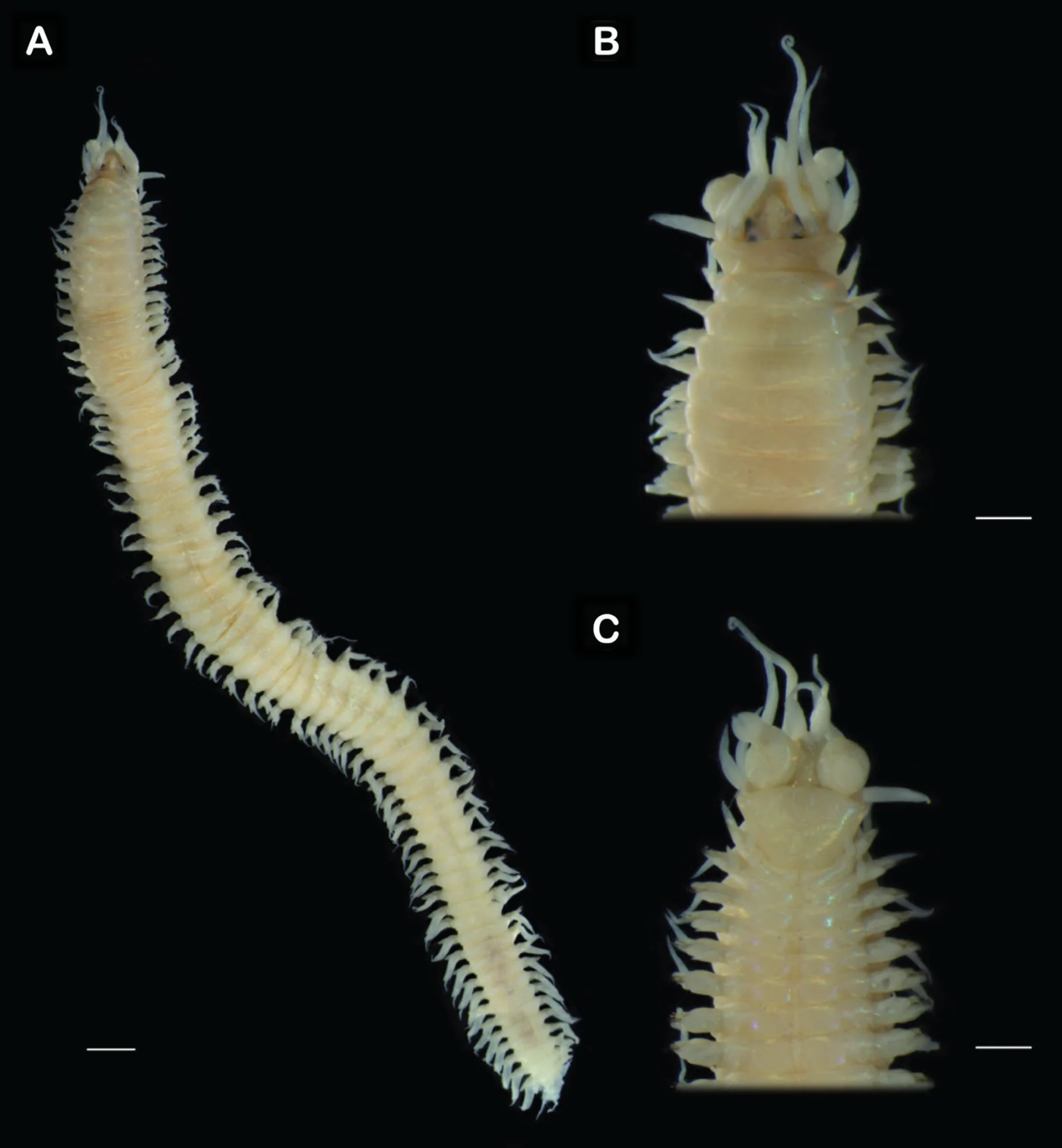
*Cheilonereis* sp. **A**. Whole body; **B**. Anterior end (dorsal view); **C**. Anterior end (ventral view). Scale bars: 2 mm (**A**); 1 mm (**B, C**).

##### Diagnosis.

Body color in alcohol cream or whitish, lacking pigmentation. Prostomium with entire anterior margin. Four circular black eyes present, arranged rather obliquely with distance between anterior eyes slightly wider than that of posterior ones. Antennae subulate. Palps biarticulated with massive subconical palpophores and globular palpostyles. Peristomium tapering posteriorly. Four pairs of smooth peristomial cirri with distinct cirrophores present; longest cirri reaching chaetiger 5. Ventral peristomial flap present. Conical paragnaths present on both rings. Prechaetal notopodial lobe restricted to anterior chaetigers. Dorsal notopodial ligule markedly broader and elongate on posterior chaetigers.

##### Distribution and habitat.

The species was obtained from Maluku, Indonesia, inhabiting intertidal rocky shore.

##### Remarks.

Our specimens fit the minimal generic diagnosis of Wilson et al. (2023), i.e., ‘ventral peristomial flap present; Areas VII–VIII conical paragnaths differentiated, with a separate band of minute paragnaths also present; dorsal notopodial ligule markedly broader on posterior chaetigers.’ To date, there are only three *Cheilonereis* species in the world, i.e., *Cheilonereis cyclurus* (Harrington, 1897) from Puget Sound (USA), *Cheilonereis peristomialis* Benham, 1916 from Great Australian Bight (Australia), and *Cheilonereis shishidoi* (Izuka, 1912) from Japan. All of these species were collected from temperate regions. The *Cheilonereis* sp. in the present study is the first material obtained from the tropical region and may represent a new species. In such a case, the species will be described in a separate taxonomic work, preferably with genetic data.

#### Composetia marmorata

Taxon classificationAnimaliaPhyllodocidaNereididae

(Horst, 1924)

005167E7-6C7B-5B99-9BA9-6409A320B2C4

Fig. 5A–D

Nereis (Ceratonereis) marmorata Horst, 1924: 177–178, pl. 34, figs 13–16.
Composetia marmorata . – Pamungkas and Glasby 2015: 8–9, fig. 3C, D.

##### Type locality.

Between Geser and Seram Islands, Maluku Province, Indonesia.

**Figure 5.**
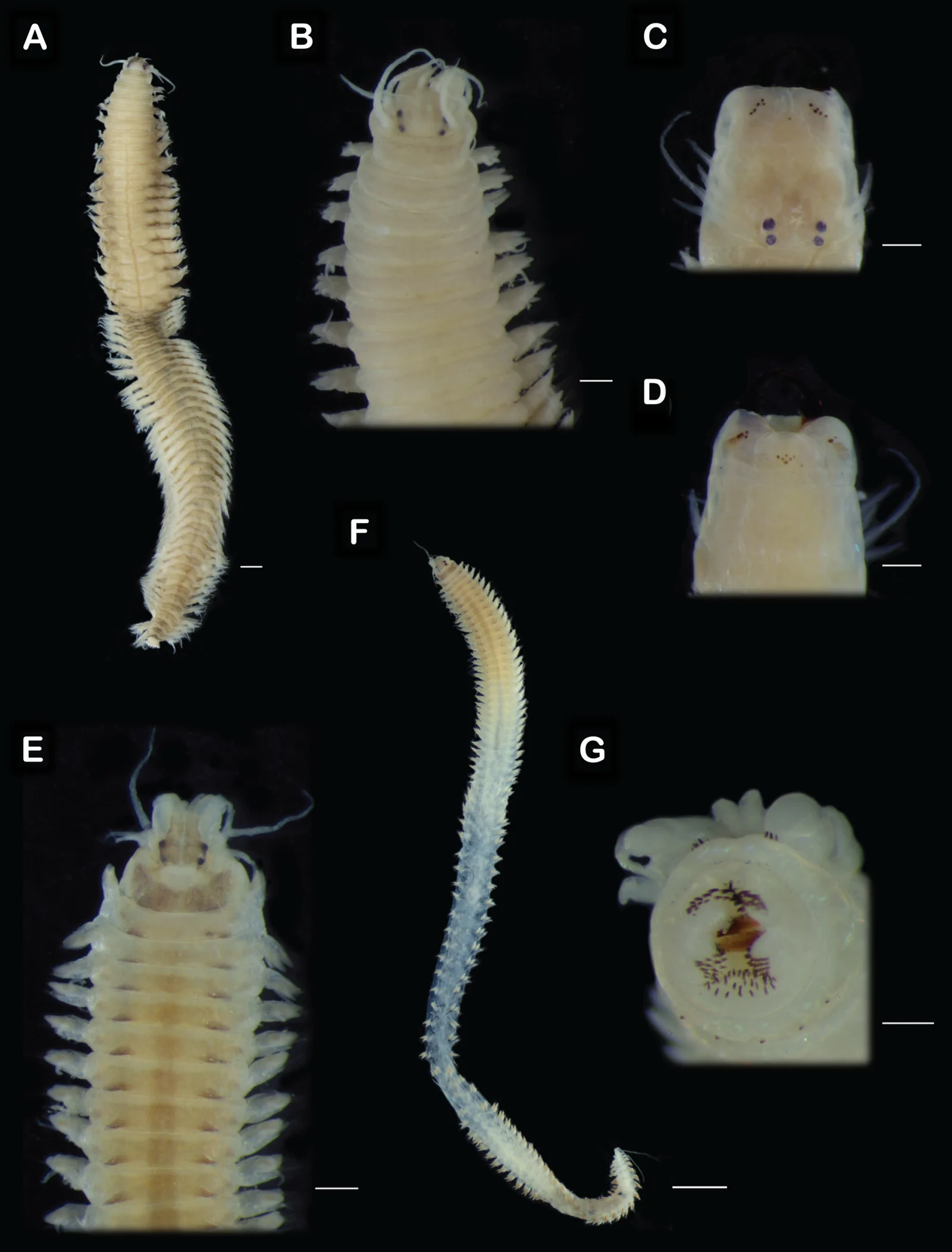
*Composetia marmorata*. **A**. Whole body of an epitoke; **B**. Anterior end of an atoke (dorsal view); **C**. Prostomium with everted proboscis (dorsal view); **D**. Prostomium with everted proboscis (ventral view); **E**. *Hediste* sp. (dorsal view of anterior end); **F**. Whole body; **G**. Everted proboscis. Scale bars: 1 mm (**A**); 0.5 mm (**B–D, F**); 2 mm (**E**); 0.25 mm (**G**).

##### Material examined.

MZB. Pol. 00314 (7 males) and MZB. Pol. 00315 (1 female), collected as heteronereids locally known as ‘*nyale*’ by Dewi C. Murniati on 8 March 2018 from Lombok Island: Seger Beach, Kuta Village, Pujut District, Central Lombok Regency, West Nusa Tenggara Province (8°54'25.9"S, 116°17'49.3"E); SMF 33463 (3 specimens), collection details same as MZB. Pol. 00314; MZB. Pol. 00276 (13 specimens), collected by Joko Pamungkas on 23 November 2017 from Warmar Island: Siwalima Village, Aru Islands District, Aru Islands Regency, Maluku Province (5°45'26.9"S, 134°13'23.5"E); MZB. Pol. 00277 (1 specimen), collected by Joko Pamungkas on 21 November 2017 from Ambon Island: Hutumuri Village, South Leitimur District, Ambon City, Maluku Province (3°41'46.1"S, 128°17'49.0"E); MZB. Pol. 00339 (2 specimens), collected by Joko Pamungkas on 24 November 2017 from Warmar Island: Wangel Village, Aru Islands District, Aru Islands Regency, Maluku Province (5°45'05.1"S, 134°11'43.2"E).

##### Comparative material.

*Composetia marmorata* as heteronereids locally known as ‘*laor/wawo*’. MZB. Pol. 00161 (3 males), MZB. Pol. 00162 (2 females), RCLA. Ann. 042 (3 males), RCLA. Ann. 043 (1 female), RCLA. Ann. 044 (3 males), RCLA. Ann. 045 (2 females), collected by Robert Alik on 18–19 March 2014 from Ambon Island: Suli Village, Salahutu District, Central Maluku Regency, Maluku Province (3°37'38.2"S, 128°18'25.0"E); RCLA. Ann. 046 (1 male), collected by Abraham S. Leatemia on 19 March 2014 from Ambon Island: Mahia Subvillage, Urimessing Village, Nusaniwe District, Ambon City, Maluku Province (3°44'42.6"S, 128°11'24.6"E).

##### Diagnosis.

Body color in alcohol cream with distinctive marmorated stripes on prostomium and anterior body. All paragnaths conical, arranged as follows. Area I = 1–2; Area II = 7–14; Area III = 9–13; Area IV = 14–16; Areas V–VIII absent. Male and female epitokes morphologically similar, yet males with sub-distally swollen (not scalloped) dorsal cirri in natatory region (unmodified in female) and pygidial rosette (lacking in females). Pre-natatory region comprising 17 or 18 chaetigers with basally swollen dorsal cirri on chaetigers 1–6 or 7, and basally swollen ventral cirri on chaetigers 1–5. Natatory region extending to pygidium. Females with unpigmented eggs (Pamungkas and Glasby 2015).

##### Distribution and habitat.

The species was found in Maluku and West Nusa Tenggara (Indonesia) and Lizard Island (NE Australia), inhabiting intertidal rocky shore.

##### Remarks.

Although the distinctive marmorated (= veined) stripes of *Composetia marmorata* appear to be faded on the present specimens, the paragnath counts match those described by Pamungkas and Glasby (2015) for Ambonese specimens and Horst’s (1924) original description. In addition, the account of *Composetia marmorata* swarming as *nyale* on Lombok Island agrees well with the description of the species swarming as *laor*/*wawo* on Ambon Island. The non-reproductive stage specimens obtained in the present work, as well as *nyale*, complement the collection of this species at MZB.

#### Hediste

Taxon classificationAnimaliaPhyllodocidaNereididae

sp.

39D15E08-4C22-58B6-8FAA-BFD69D235D05

Fig. 5E–G

##### Material examined.

MZB. Pol. 00278 (4 specimens), collected by Joko Pamungkas on 23 November 2017 from Warmar Island: Siwalima Village, Aru Islands District, Aru Islands Regency, Maluku Province (5°45'26.9"S, 134°13'23.5"E).

##### Diagnosis.

Body color in alcohol cream with striking brown pigmentation in some areas, including prostomium (except around midline), peristomium (as a transverse band), dorsal area of chaetigers 1–16 (as a longitudinal band around midline), and lateral side of dorsal area of same chaetigers (as a bar on each side). Prostomium with entire anterior margin. Four circular black eyes present, arranged rather obliquely with distance between anterior eyes slightly wider than that of posterior ones. Antennae conical. Palps biarticulated with subconical palpophores and elongated palpostyles. Peristomium length greater than that of chaetiger 1. Four pairs of peristomial cirri with distinct cirrophores present; longest cirri reaching chaetiger 6. Paragnaths present on both rings; Area VI without smooth-bar paragnaths. Dorsal fascicle of neurochaetae with heterogomph falcigers (in anterior chaetae) and simple (fused) falcigers.

##### Distribution and habitat.

The species was obtained from Maluku, Indonesia, inhabiting intertidal sandy shores with reef rubble.

##### Remarks.

Our specimens fit the minimal generic diagnosis of Wilson et al. (2023), i.e., ‘Neurochaetae dorsal fascicle simple chaetae (fused falcigers) present; palpophore massive subconical, flattened (palpostyle is minute by comparison)’. *Hediste* is one of the least species-rich nereidid genera with only eight species described worldwide. Namely, *Hediste astae* Teixeira et al., 2022, *Hediste atoka* Sato & Nakashima, 2003, *Hediste diadroma* Sato & Nakashima, 2003, *Hediste diversicolor* (O.F. Müller, 1776), *Hediste japonica* (Izuka, 1908), *Hediste limnicola* (Johnson, 1903), *Hediste pontii* Teixeira et al., 2022, *Hediste rabatensis* (Mohammad, 1989). *Hediste* sp. in the present work may represent a new species. In such a case, the species will be described in a separate taxonomic work, preferably with genetic data.

#### Neanthes cf. gisserana

Taxon classificationAnimaliaPhyllodocidaNereididae

(Horst, 1924)

73550C3B-1E35-5DD8-B720-C02E8B2B0CF9

Fig. 6A–C

Nereis (Lycoris) gisserana Horst, 1924: 151–152, pl. 30, figs 6, 7.
Nereis gisserana . – Monro 1939, fig. 302a–f.
Neanthes sp. cf. N. gisserana. – Pamungkas and Glasby 2015: 11–12, fig. 4C, D.

##### Material examined.

MZB. Pol. 00312 (106 males) and MZB. Pol. 00313 (59 females), collected as heteronereids locally known as ‘*nyale*’ by Dewi C. Murniati on 8 March 2018 from Lombok Island: Seger Beach, Kuta Village, Pujut District, Central Lombok Regency, West Nusa Tenggara Province (8°54'25.9"S, 116°17'49.3"E). SMF 33464 (5 males) and SMF 33465 (4 females), collection details as for MZB Pol. 00312 and MZB Pol. 00313, respectively.

**Figure 6.**
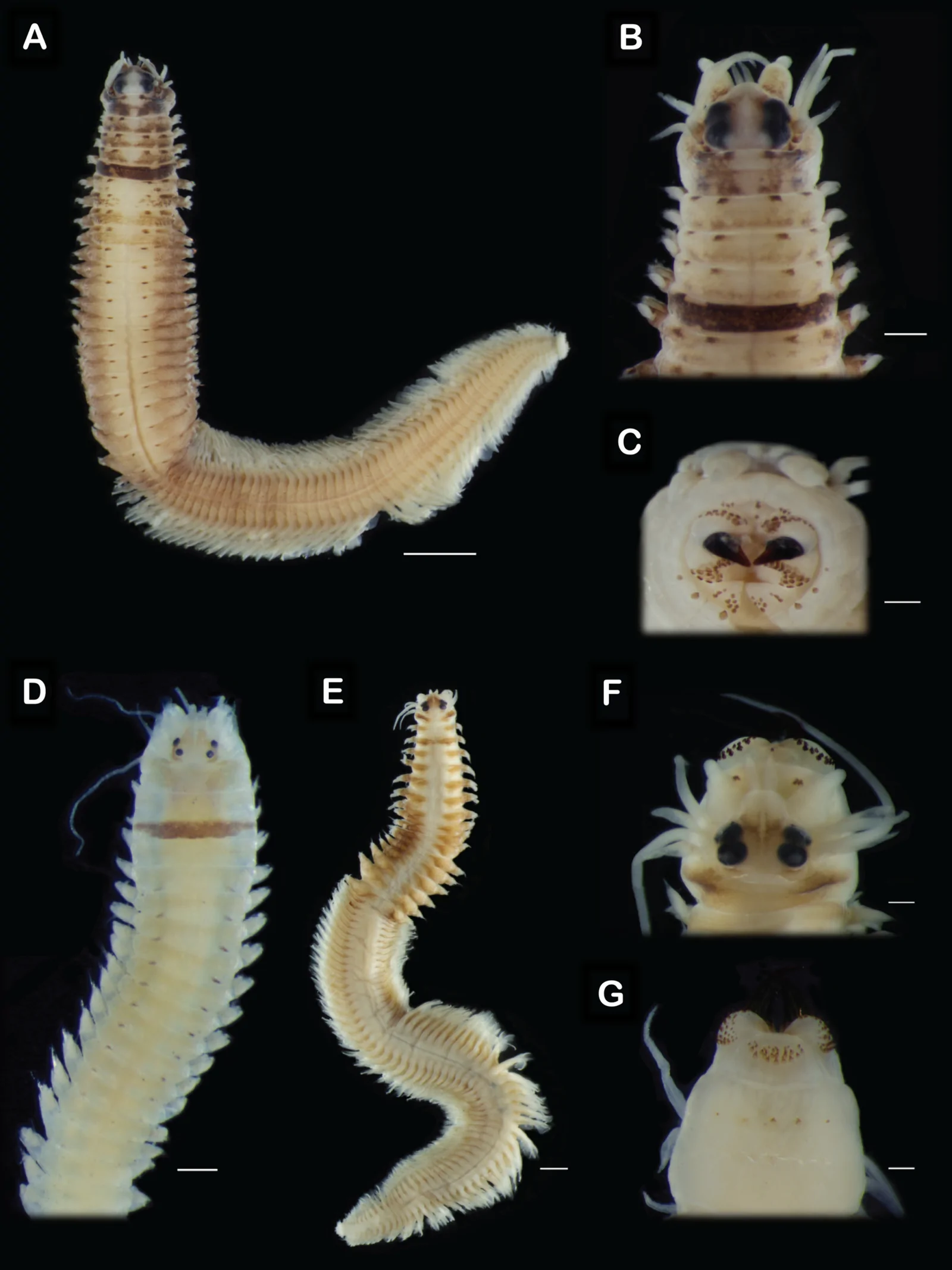
*Neanthes* species. **A**. Neanthes cf. gisserana (whole body of an epitoke); **B**. Anterior end (dorsal view); **C**. Everted proboscis; **D**. *Neanthes unifasciata* (anterior end of an atoke); **E**. Whole body of an epitoke; **F**. Prostomium with everted proboscis (dorsal view); **G**. Prostomium with everted proboscis (ventral view). Scale bars: 2 mm (**A**); 0.5 mm (**B–D**); 1 mm (**E**); 0.25 mm (**F, G**).

##### Comparative material.

*Neanthes* sp. cf. *N. gisserana* as heteronereids locally known as ‘*laor/wawo*’. MZB. Pol. 00167 (2 males), MZB. Pol. 00168 (2 females), RCLA. Ann. 031 (5 males), RCLA. Ann. 032 (3 females), RCLA. Ann. 033 (1 male) and RCLA. Ann. 034 (1 female), collected by Robert Alik on 18–19 March 2014 from Ambon Island: Suli Village, Salahutu District, Central Maluku Regency, Maluku Province (3°37'38.2"S, 128°18'25.0"E); RCLA. Ann. 035 (3 males), RCLA. Ann. 036 (2 females) and RCLA. Ann. 037 (2 males), collected by Abraham S. Leatemia on 18–19 March 2014 from Ambon Island: Mahia Subvillage, Urimessing Village, Nusaniwe District, Ambon City, Maluku Province (3°44'42.6"S, 128°11'24.6"E).

##### Diagnosis.

Body color in alcohol cream with a striking dark brown band on dorsum of chaetiger 4. Paragnaths consisting of cones and bars arranged as follows. Area I = 4 (in a longitudinal line); Area II = 14; Area III = 19–22 (including lateral groups); Area IV = 26 (includes cones and bars); Area V = 0; Area VI = 3–6; Areas VII–VIII = 5–7 (in a transverse line). Males and females showing similar body pigmentation and natatory region extending to pygidium, yet different in numbers of pre-natatory chaetigers and modified dorsal and ventral cirri. Males with pre-natatory region comprising 21 or 22 chaetigers. Basally swollen dorsal and ventral cirri observed on chaetigers 1–7 and 1–5 or 6, respectively. Females with pre-natatory region comprising 26 chaetigers. Basally swollen dorsal and ventral cirri observed on chaetigers 1–6 and 1–5, respectively. Males with pygidial rosette and sub-distally swollen (not scalloped) dorsal cirri, lacking in females (Pamungkas and Glasby 2015).

##### Distribution and habitat.

The species was found in Maluku and West Nusa Tenggara (Indonesia) and Lizard Island (NE Australia), inhabiting intertidal rocky shore.

##### Remarks.

Neanthes cf. gisserana swarming as *nyale* on Lombok Island agrees well with the description of the species swarming as *laor/wawo* on Ambon Island referred to as *Neanthes* sp. cf. *N. gisserana* by Pamungkas and Glasby (2015). They are also very similar to the species collected from Lizard Island, northern Great Barrier Reef, Australia, identified by Glasby (2015). The worms possibly represent a new species and are likely to inhabit subtidal areas as the non-reproductive stage of this species has not been found in Indonesia. The non-reproductive stage is needed to describe this species.

#### Neanthes unifasciata

Taxon classificationAnimaliaPhyllodocidaNereididae

(Willey, 1905)

F2D97EE9-089B-5F8E-936C-4F80C191D47E

Fig. 6D–G

Nereis unifasciata Willey, 1905: 271–272, Pl. IV, figs 85–88.
Neanthes unifasciata . – Pamungkas and Glasby 2015: 9–11, fig. 4A, B.

##### Type locality.

Gulf of Mannar, Sri Lanka.

##### Material examined.

MZB. Pol. 00282 (3 specimens), collected by Joko Pamungkas on 23 November 2017 from Warmar Island: Siwalima Village, Aru Islands District, Aru Islands Regency, Maluku Province (5°45'26.9"S, 134°13'23.5"E); MZB. Pol. 00310 (41 males) and MZB. Pol. 00311 (20 females), collected as heteronereids locally known as ‘*nyale*’ by Dewi C. Murniati on 8 March 2018 from Lombok Island: Seger Beach, Kuta Village, Pujut District, Central Lombok Regency, West Nusa Tenggara Province (8°54'25.9"S, 116°17'49.3"E). SMF 33466 (4 males) and SMF 33467 (2 females), collection details as for MZB Pol. 00310 and MZB Pol. 00311, respectively.

##### Comparative material.

*Neanthes unifasciata* as heteronereids locally known as ‘*laor/wawo*’. RCLA. Ann. 020 (4 males) and RCLA. Ann. 021 (6 females), collected by Joko Pamungkas on 19 March 2014 from Ambon Island: Allang Village, West Leihitu District, Central Maluku Regency, Maluku Province (03°46'18.2"S, 128°00'24.6"E); RCLA. Ann. 022 (10 males) and RCLA. Ann. 023 (11 females), collected by Joko Pamungkas on 19 March 2014 from Ambon Island: Lilibooi Village, West Leihitu District, Central Maluku Regency, Maluku Province (03°45'08.8"S, 128°01'24.6"E); MZB. Pol. 00170 (1 male), RCLA. Ann. 024 (111 males), RCLA. Ann. 025 (57 females) and RCLA. Ann. 026 (2 females), collected by Robert Alik on 18–19 March 2014 from Ambon Island: Suli Village, Salahutu District, Central Maluku Regency, Maluku Province (3°37'38.2"S, 128°18'25.0"E); MZB. Pol. 00169 (3 males), RCLA. Ann. 027 (38 males), RCLA. Ann. 028 (41 females), RCLA. Ann. 029 (6 males) and RCLA. Ann. 030 (5 females), collected by Abraham S. Leatemia on 18–19 March 2014 from Ambon Island: Mahia Subvillage, Urimessing Village, Nusaniwe District, Ambon City, Maluku Province (3°44'42.6"S, 128°11'24.6"E).

##### Diagnosis.

Body color in alcohol cream with dark brown band on dorsum of chaetiger 2, and lighter bands on following pre-natatory chaetigers (in epitokes). All paragnaths conical, arranged as follows. Area I = 1; Area II = 14–16; Area III = 22–25; Area IV = 25–30; Area V = 0; Area VI = 6–12 (very small); Area VII–VIII = 4–6 in one line. Both male and female epitokes with natatory region extending to pygidium, yet different in number of pre-natatory chaetigers and number of modified dorsal and ventral cirri. Males having pre-natatory region comprising 16 chaetigers with basally swollen dorsal cirri on chaetigers 1–7 and basally swollen ventral cirri on chaetigers 1–5 or 6. Females having pre-natatory region comprising 19 or 20 chaetigers with basally swollen dorsal and ventral cirri on chaetigers 1–5. Males different from females in having subdistally swollen and scalloped dorsal cirri (smooth in females) and modified rosette pygidium (Pamungkas and Glasby 2015).

##### Distribution and habitat.

The species was found in Indo-West Pacific, inhabiting intertidal rocky shores.

##### Remarks.

*Neanthes unifasciata* swarming as *nyale* on Lombok Island agrees well with the species swarming as *laor/wawo* on Ambon Island and is one of the most common heteronereids. In the present specimens, the color intensity of the brown bands on the dorsal surface of pre-natatory chaetigers is similar (Fig. 6E). The non-reproductive stage specimens obtained in the present work, as well as *nyale*, complement the collection of this species at MZB.

#### Neanthes

Taxon classificationAnimaliaPhyllodocidaNereididae

sp. 1

19B30D50-6D7A-5899-B4A7-C896DAFB925C

Fig. 7A

##### Material examined.

MZB. Pol. 00281 (1 female), collected by Joko Pamungkas on 24 November 2017 from Warmar Island: Wangel Village, Aru Islands District, Aru Islands Regency, Maluku Province (5°45'05.1"S, 134°11'43.2"E).

**Figure 7.**
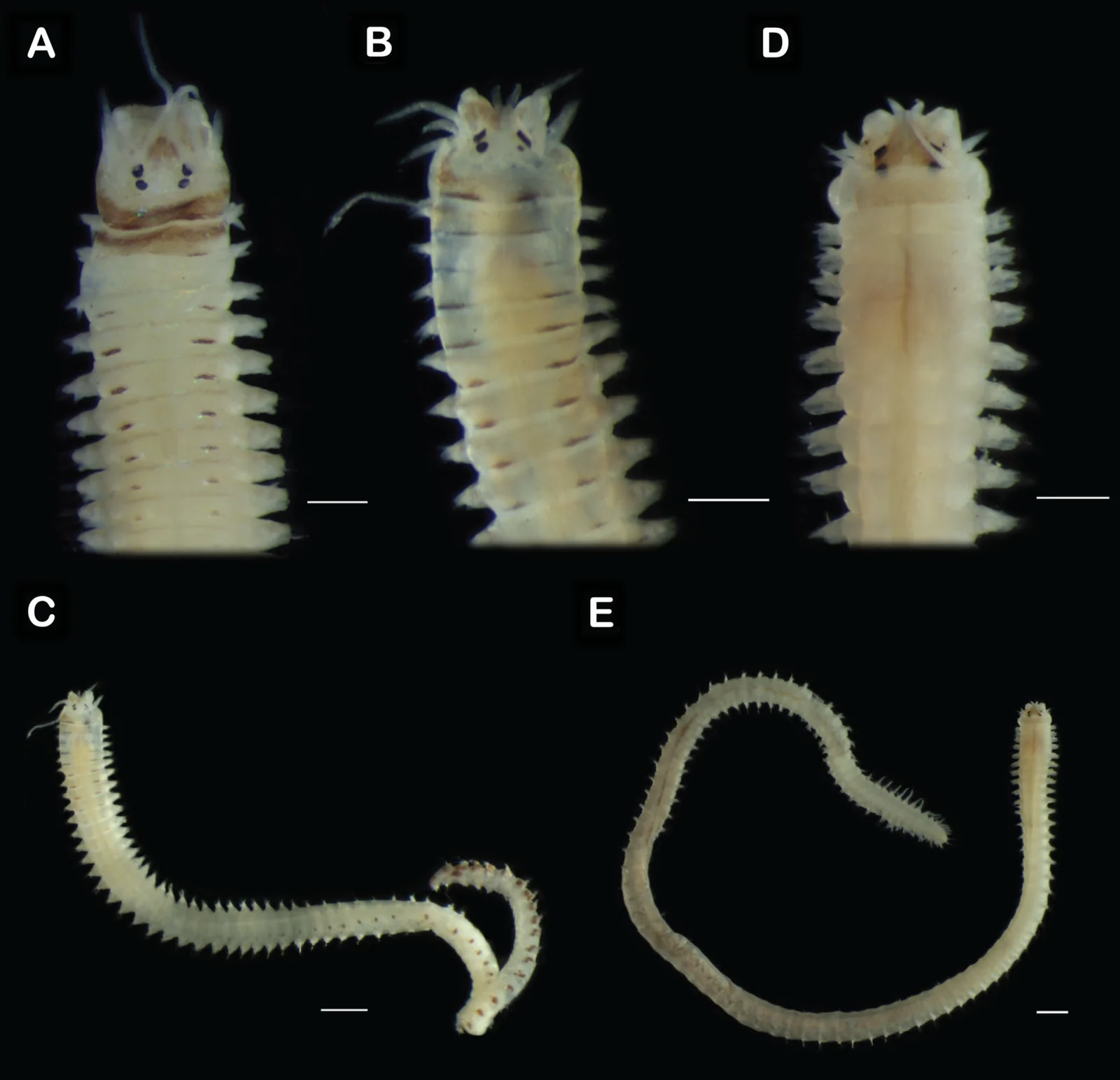
*Neanthes* species. **A**. *Neanthes* sp. 1 (dorsal view of anterior end); **B**. *Neanthes* sp. 2 (dorsal view of anterior end); **C**. Whole body; **D**. *Neanthes* sp. 3 (dorsal view of anterior end); **E**. Whole body. Scale bars: 0.5 mm (**A, B, D**); 1 mm (**C, E**).

##### Diagnosis.

Single epitokous specimen incomplete. Body color in alcohol cream with brown pigmentation in some areas, including anterior part of prostomium (brown triangular), dorsum of peristomium and chaetiger 1 (brown band), and dorsum of subsequent anterior chaetigers (a brown bar on each lateral side). Prostomium with entire anterior margin. Four circular black eyes present, arranged obliquely with distance between anterior eyes wider than that of posterior ones. Antennae conical. Palps biarticulated with barrel-shaped palpophores and rounded palpostyles. Peristomium bearing four pairs of smooth peristomial cirri with distinct cirrophores; longest cirri reaching chaetiger 3. Conical paragnaths present on both rings. Notoaciculae of chaetigers 1 and 2 absent. Notochaetae without homogomph falcigers.

##### Distribution and habitat.

The species was obtained from Maluku, Indonesia, inhabiting intertidal sandy shore with reefs.

##### Remarks.

*Neanthes* species with brown bars on the dorsum of anterior segments are common in Indonesian waters (and the wider Indo-West Pacific), and although the color patterns of the present species may turn out to be diagnostic, more specimens, especially non-reproductive forms, are required to assess the variation in this and other morphological features.

#### Neanthes

Taxon classificationAnimaliaPhyllodocidaNereididae

sp. 2

BC2239AC-4D8A-5080-8209-D95564CB867F

Fig. 7B, C

##### Material examined.

MZB. Pol. 00324 (1 female), collected by Joko Pamungkas on 15 December 2017 from Lembeh Island: Mawali Urban Village, North Lembeh District, Bitung City, North Sulawesi Province (1°26'35.3"N, 125°13'48.9"E).

##### Diagnosis.

Single epitokous specimen incomplete. Body color in alcohol cream with a single brown bar on each lateral side of anterior dorsal segments. Prostomium with entire anterior margin. Four black eyes present; anterior eyes roughly rectangular; posterior eyes circular. Antennae subulate. Palps biarticulated with barrel-shaped palpophores and rounded palpostyles. Peristomium bearing four pairs of smooth peristomial cirri with distinct cirrophores; longest cirri reaching chaetiger 5. Conical paragnaths present on both rings. Notoaciculae of chaetigers 1 and 2 absent. Notochaetae without homogomph falcigers.

##### Distribution and habitat.

The species was obtained from North Sulawesi, Indonesia, inhabiting intertidal rocky shore.

##### Remarks.

More specimens, especially non-reproductive forms, are required to further identify the present *Neanthes* species.

#### Neanthes

Taxon classificationAnimaliaPhyllodocidaNereididae

sp. 3

974A5795-E52E-5FAD-926E-B7C0AEECD655

Fig. 7D, E

##### Material examined.

MZB. Pol. 00325 (4 specimens), collected by Joko Pamungkas on 28 August 2015 from Ambon Island: Poka Village, Teluk Ambon District, Ambon City, Maluku Province (3°38'45.3"S, 128°11'31.6"E); MZB. Pol. 00326 (5 specimens), collected by Joko Pamungkas on 3 September 2015 from Ambon Island: Negeri Lama Village, Teluk Ambon Baguala District, Ambon City, Maluku Province (3°37'47.5"S, 128°14'09.6"E).

##### Diagnosis.

Body color in alcohol cream, slightly tapering to approximately chaetiger 10, then remaining roughly uniform in width posteriorly. Prostomium with entire anterior margin. Four circular black eyes present; anterior and posterior eyes arranged in a longitudinal row. Antennae conical. Palps biarticulated with subconical palpophores and rounded palpostyles. Peristomium with convex lateral margin. Four pairs of smooth short peristomial cirri with distinct cirrophores present; longest cirri reaching chaetiger 1. Conical paragnaths present on both rings. Notoaciculae of chaetigers 1 and 2 absent. Notochaetae without homogomph falcigers.

##### Distribution and habitat.

The species was obtained from Maluku, Indonesia, inhabiting mangroves.

##### Remarks.

Further identification is required to reveal the identity of the present *Neanthes* specimens (comparative material is ideally needed).

#### Perinereis helleri

Taxon classificationAnimaliaPhyllodocidaNereididae

(Grube, 1878)

D60D8794-9768-5133-8744-AB74BCFAACB7

Fig. 8A–D

Nereis (Perinereis) helleri Grube, 1878: 81–82.
Perinereis cultrifera . – Martens et al. 1995: 15 (non Grube).
Perinereis helleri . – Hutchings et al. 1991: 254–255, fig. 9a–c. Pamungkas and Glasby 2015: 15–17, fig. 6A, B.

##### Type locality.

Bohol, Philippines.

**Figure 8.**
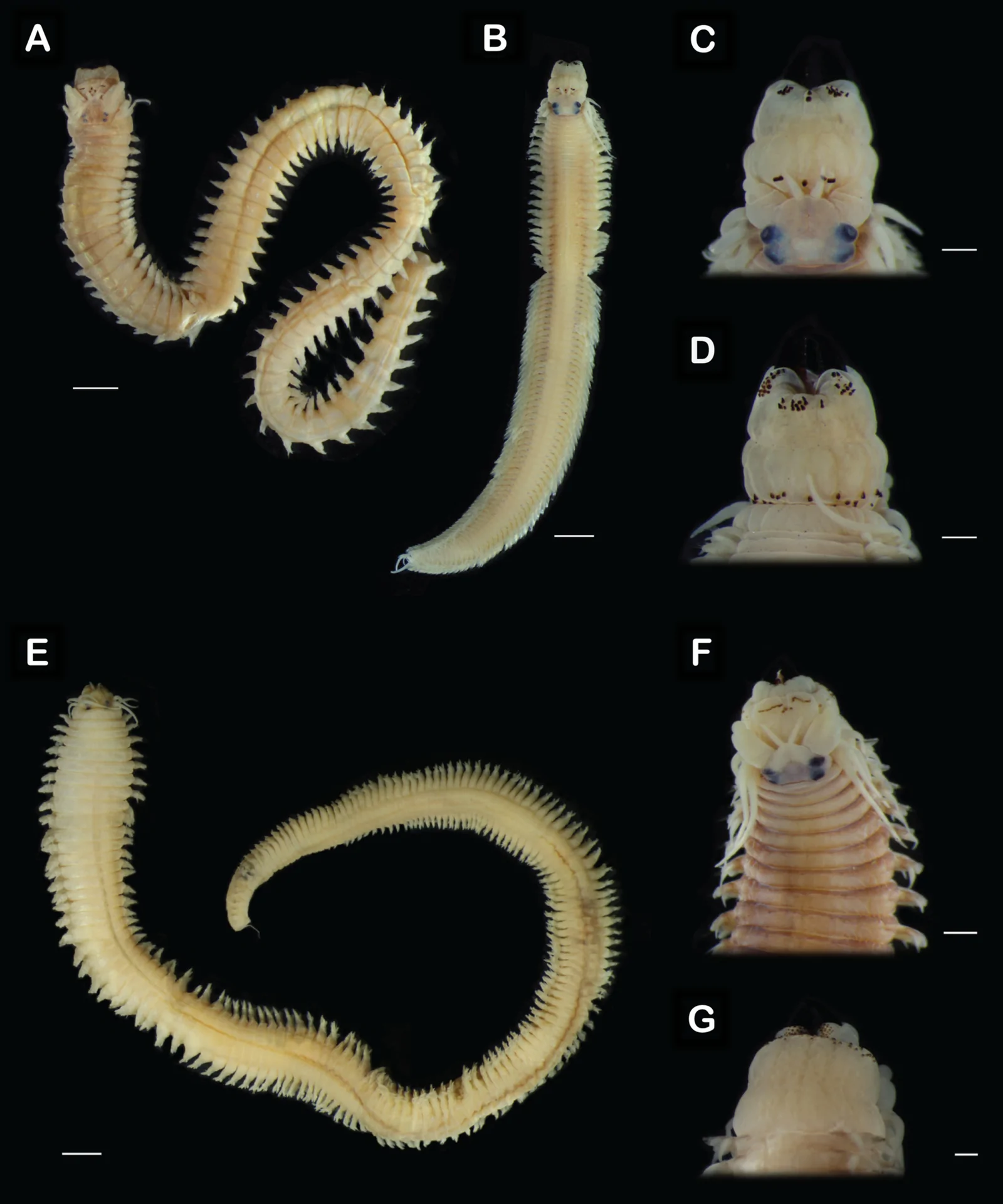
*Perinereis* species. **A**. *Perinereis helleri* (atoke); **B**. Epitoke; **C**. Prostomium with everted proboscis (dorsal view); **D**. Prostomium with everted proboscis (ventral view); **E**. *Perinereis larentukana* (whole body); **F**. Anterior end with everted proboscis (dorsal view); **G**. Prostomium with everted proboscis (ventral view). Scale bars: 2 mm (**A, B, E**); 0.5 mm (**C, D, G**); 1 mm (**F**).

##### Material examined.

MZB. Pol. 00283 (1 specimen), collected by Joko Pamungkas on 15 December 2017 from Lembeh Island: Mawali Urban Village, North Lembeh District, Bitung City, North Sulawesi Province (1°26'35.3"N, 125°13'48.9"E); MZB. Pol. 00284 (1 specimen), collected by Joko Pamungkas on 24 November 2017 from Warmar Island: Wangel Village, Aru Islands District, Aru Islands Regency, Maluku Province (5°45'05.1"S, 134°11'43.2"E); MZB. Pol. 00285 (2 specimens), collected by Joko Pamungkas on 21 November 2017 from Ambon Island: Hutumuri Village, South Leitimur District, Ambon City, Maluku Province (3°41'46.1"S, 128°17'49.0"E); MZB. Pol. 00297 (8 specimens), collected by Joko Pamungkas on 19 December 2017 from Barrang Lompo Island: Barrang Lompo Village, Sangkarrang Island District, Makassar City, South Sulawesi Province (5°02'45.7"S, 119°19'43.4"E); MZB. Pol. 00301 (80 males) and MZB. Pol. 00302 (63 females), collected as heteronereids locally known as *nyale* by Geterudis Kerans in March 2022 from Sumba Island: Pero Batang Village, Kodi District, Southwest Sumba Regency, East Nusa Tenggara Province (9°36'27.7"S, 118°59'06.0"E). SMF 33468 (1 male), collection details as for MZB. Pol. 00301.

##### Comparative material.

*Perinereis helleri* as heteronereids locally known as ‘*laor/wawo*’. RCLA. Ann. 001 (4 males), RCLA. Ann. 002 (2 females), collected by Joko Pamungkas on 19 March 2014 from Ambon Island: Allang Village, West Leihitu District, Central Maluku Regency, Maluku Province (03°46'18.2"S, 128°00'24.6"E); RCLA. Ann. 003 (6 males), RCLA. Ann. 004 (6 females), collected by Eduard Moniharapon on 19 March 2014 from Ambon Island: Hutumuri Village, South Leitimur District, Ambon City, Maluku Province (03°41'27.5"S, 128°17'56.3"E); RCLA. Ann. 005 (1 female), collected by Abraham S. Leatemia on 18 March 2014 from Ambon Island: Mahia Subvillage, Urimessing Village, Nusaniwe District, Ambon City, Maluku Province (3°44'42.6"S, 128°11'24.6"E); MZB. Pol. 00163 (5 males), MZB. Pol. 00164 (5 females), RCLA. Ann. 006 (11 males) and RCLA. Ann. 007 (10 females), collected by Franky E. de Soysa on 19 March 2014 from Ambon Island: Subvillage Airlouw, Nusaniwe Village, Nusaniwe District, Ambon City, Maluku Province (03°46'32.5"S, 128°07'53.5"E). *Perinereis helleri*. ZMH-ANN-P16551 (2 specimens), collected from Western Australia. *Perinereis cultrifera*. ZMH-ANN-P13796 (10 specimens), collected from Bermuda.

##### Diagnosis.

Body color in alcohol cream with brown streaky pigmentation on dorsum. Pharynx with conical paragnaths on both rings, smooth bars present on Areas VI, arranged as follows. Area I = 2–3; Area II = 6–10; Area III = 14–18 (including lateral groups); Area IV = 17–24; Area V = 3; Area VI = 1 bar; Area VII–VIII = 25–30. Male and female epitokes have similar body pigmentation and natatory region extending to pygidium, but differ in number of pre-natatory chaetigers and number of modified dorsal and ventral cirri. Males have pre-natatory region comprising 17 chaetigers with basally swollen dorsal and ventral cirri on chaetigers 1–7 and 1–6, respectively. Females have pre-natatory region comprising 19–20 chaetigers with basally swollen dorsal and ventral cirri on chaetigers 1–6 and 1–5, respectively. In addition, males have pygidial rosette and scalloped dorsal cirri, lacking in female (Pamungkas and Glasby 2015).

##### Distribution and habitat.

The species was found in Indo-Pacific, inhabiting intertidal rocky shores.

##### Remarks.

*Perinereis helleri* swarming as *nyale* on Sumba Island agrees well with the species swarming as *laor/wawo* on Ambon Island, and is one of the most common *nyale*/*laor*/*wawo* species. The paragnath arrangement of this species is very similar to that of *P. cultrifera*, which Martens et al. (1995) apparently mistook it for. The morphology of the reproductive stage of this species is similar to that of *Perinereis nigropunctata*. The non-reproductive stage specimens obtained in the present work, as well as *nyale*, complement the collection of this species at MZB.

#### Perinereis larentukana

Taxon classificationAnimaliaPhyllodocidaNereididae

(Grube, 1881)

984B994A-DC63-589C-A6F5-1365B6165CB1

Fig. 8E–G

Nereis larentukana Grube, 1881: 110–111.
Neanthes larentukana . – Hartman 1959: 250. Wiktor 1980: 273.
Nereis larentukana . – Hartwich 1993: 111.
Perinereis larentukana comb. nov. by Villalobos-Guerrero 2019: 483–488, figs 1G–I, 8A–L, 9A–F.

##### Type locality.

East Nusa Tenggara, Indonesia.

##### Material examined.

MZB. Pol. 00246 (9 specimens), collected by Joko Pamungkas on 23 March 2015 from Saparua Island: Saparua Village, Saparua District, Central Maluku Regency, Maluku Province (3°34'24.9"S, 128°39'26.7"E); SMF 33469 (2 specimens), collection details as in MZB. Pol. 00246. MZB. Pol. 00249 (13 specimens), collected by Joko Pamungkas on 5 October 2009 from Nusalaut Island: Akoon Village, Nusalaut District, Central Maluku Regency, Maluku Province (3°40'16.3"S, 128°48'35.1"E); MZB. Pol. 00303 (55 specimens), collected by Geterudis Kerans on 20 April 2024 from Pero Batang Village, Kodi District, Southwest Sumba Regency, East Nusa Tenggara Province (9°36'26.8"S, 118°59'07.1"E); MZB. Pol. 00307 (6 males) and MZB. Pol. 00308 (1 female), collected as heteronereids locally known as *nyale* by Dewi C. Murniati on 8 March 2018 from Lombok Island: Seger Beach, Kuta Village, Pujut District, Central Lombok Regency, West Nusa Tenggara Province (8°54'25.9"S, 116°17'49.3"E).

##### Comparative material.

*Perinereis nuntia*. ZMH-ANN-P18075 (2 specimens).

##### Diagnosis.

Prostomium with antennae joined medially and nuchal organs three times longer than posterior eyes. Postero-dorsal tentacular cirri reaching chaetigers 2–3. Pharynx with conical paragnaths on both rings, smooth bars present on Areas VI, arranged as follows. Area I = 2–3; Area II = 7–9; Area III = 9–17, 1 lateral cone; Area IV = 20–28, merged paragnaths absent; Area V = 3–4, paragnaths behind Area VI; Area VI = 4–8, arc short, regular, bars short, even, except inner one slightly longer; Areas VI–V–VI pattern, υ-shaped (ridges of VI sub-medially markedly separated, parallel furrows); Areas VII–VIII = 26–34 in two transverse rows, proximal with large cones. Gap between Area VI and Areas VII–VIII broad. Dorsal cirri short, one-half length of dorsal ligule, posteriorly, inserted at two-thirds of dorsal ligule. Dorsal ligule even throughout body, subequal to median ligule; distal lobe bluntly conical posteriorly. Ventral cirri short and digitiform. Homogomph spinigers with finely serrated blades, evenly spaced, absent in subacicular neurochaetae. Heterogomph spinigers as notopodial; absent in a few anterior chaetigers. Heterogomph falcigers with short and medium blades (modified after Villalobos-Guerrero 2019).

##### Distribution and habitat.

The species was found in East Nusa Tenggara and Maluku Provinces, Indonesia, inhabiting intertidal sandy shores.

##### Remarks.

The specimens in the present study agree well with previous descriptions. The body color of *P. larentukana* from Saparua Island tends to be light maroon, whereas the species from Nusalaut Island tends to be whitish. As the species has not been reported elsewhere, *P. larentukana* appears to be endemic to Indonesia.

*Perinereis larentukana* is considered a member of *Perinereis nuntia* species complex, mainly by having conical paragnaths on both pharyngeal rings, an arc of several shield-shaped paragnaths arranged in a row on Area VI, notochaetae with only homogomph spinigers, and slightly uneven dorsal and median ligules throughout the body (Wilson and Glasby 1993; Glasby and Hsieh 2006; Villalobos-Guerrero 2019). This study is the first report of *P. larentukana* occurring among reproductive *nyale* (the species is one of the biggest *nyale* worms). The reproductive-stage specimens obtained in the present work, swarming as *nyale* worms, complement the collection of this species at MZB.

*Perinereis larentukana* appears to be one of the most common Indonesian species inhabiting sandy beaches in the eastern and western (Pari Island, off Jakarta, pers. obs. CJG) parts of the country. Because of its relatively large body size, *P. larentukana* is often utilized by the locals as fishing bait.

#### Perinereis nigropunctata

Taxon classificationAnimaliaPhyllodocidaNereididae

(Horst, 1889)

882B2595-B9B1-587D-8ABE-817142E50593

Fig. 9A–C

Nereis nigropunctata Horst, 1889: 171–174, Pl. VIII, figs 1–3.
Perinereis nigropunctata . – Hutchings et al. 1991: 256–257, fig. 10a–e. Pamungkas and Glasby 2015: 18, fig. 6C, D.

##### Type locality.

Malaysia.

**Figure 9.**
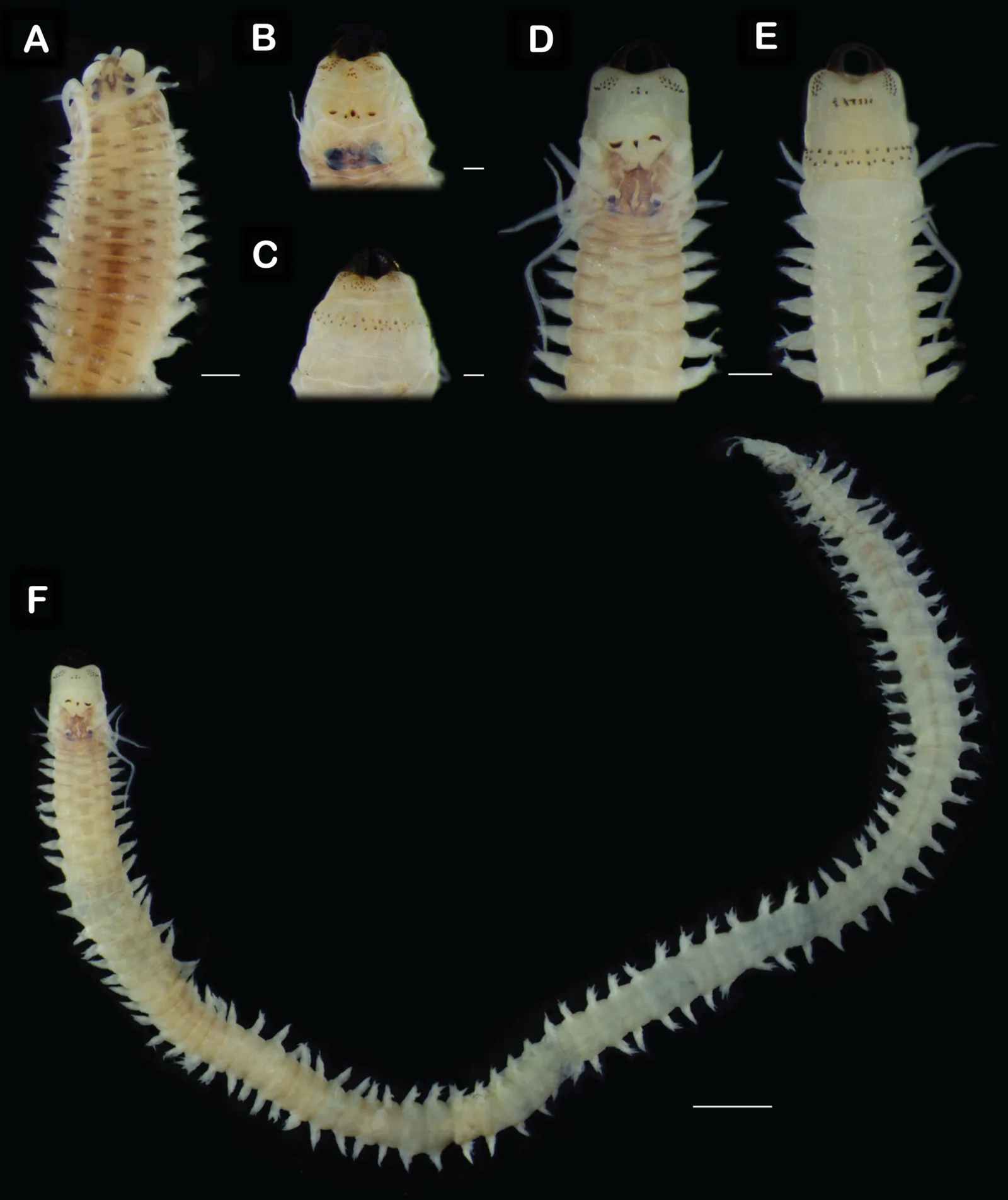
*Perinereis* species. **A**. *Perinereis nigropunctata* (dorsal view of anterior end); **B**. Prostomium with everted proboscis (dorsal view); **C**. Prostomium with everted proboscis (ventral view); **D**. *Perinereis obfuscata* (dorsal view of anterior end with everted proboscis); **E**. Anterior end with everted proboscis (ventral view); **F**. Whole body. Scale bars: 0.5 mm (**A, E, F**); 0.25 mm (**B, C**); 2 mm (**D**).

##### Material examined.

MZB. Pol. 00286 (4 specimens), collected by Joko Pamungkas on 21 November 2017 from Ambon Island: Leahari Village, South Leitimur District, Ambon City, Maluku Province (3°42'32.1"S, 128°16'10.2"E); MZB. Pol. 00290 (2 specimens), collected by Joko Pamungkas on 21 November 2017 from Ambon Island: Hutumuri Village, South Leitimur District, Ambon City, Maluku Province (3°41'46.1"S, 128°17'49.0"E); MZB. Pol. 00340 (1 specimen), collected by Joko Pamungkas on 24 November 2017 from Warmar Island: Wangel Village, Aru Islands District, Aru Islands Regency, Maluku Province (5°45'05.1"S, 134°11'43.2"E); MZB. Pol. 00341 (5 specimens), collected by Joko Pamungkas on 19 December 2017 from Barrang Lompo Island: Barrang Lompo Village, Sangkarang Island District, Makassar City, South Sulawesi Province (5°02'45.7"S, 119°19'43.4"E).

##### Comparative material.

*Perinereis nigropunctata* as heteronereids locally known as ‘*laor/wawo*’. MZB. Pol. 00165 (5 males), MZB. Pol. 00166 (3 females), RCLA. Ann. 008 (7 males), RCLA. Ann. 009 (9 females), RCLA. Ann. 010 (19 males) and RCLA. Ann. 011 (17 females), collected by Robert Alik on 18–19 March 2014 from Ambon Island: Suli Village, Salahutu District, Central Maluku Regency, Maluku Province (3°37'38.2"S, 128°18'25.0"E); RCLA. Ann. 012 (18 males), RCLA. Ann. 013 (32 females), collected by Eduard Moniharapon on 19 March 2014 from Ambon Island: Hutumuri Village, South Leitimur District, Ambon City, Maluku Province (03°41'27.5"S, 128°17'56.3"E); RCLA. Ann. 014 (1 male), RCLA. Ann. 015 (4 females), RCLA. Ann. 016 (1 male) and RCLA. Ann. 017 (1 female), collected by Abraham S. Leatemia on 18–19 March 2014 from Ambon Island: Mahia Subvillage, Urimessing Village, Nusaniwe District, Ambon City, Maluku Province (3°44'42.6"S, 128°11'24.6"E); RCLA. Ann. 018 (1 male) and RCLA. Ann. 019 (4 females), collected by Joko Pamungkas on 19 March 2014 from Ambon Island: Allang Village, West Leihitu District, Central Maluku Regency, Maluku Province (03°46'18.2"S, 128°00'24.6"E); *Perinereis nigropunctata*. ZMH-ANN-P16552 (4 specimens), collected from Western Australia.

##### Diagnosis.

Body color in alcohol cream-coffee brown with brown pigmentation on dorsum. Prostomium trapezoidal with deep median furrow anteriorly. Eyes small with distinct lenses. Palps with robust, short palpophores and globular palpostyles. Antennae short and triangular. Longest tentacular cirri extending to chaetiger 3. Peristomium relatively long. Pharynx with conical paragnaths on both rings, smooth bars present on Areas VI, arranged as follows. Area I = 4–9; Area II = 12–39 in 2 curved rows; Area III = 21–35 sometimes with two or three cones displaced laterally, separate from main group; Area IV = 18–42; Area V = 3 in triangle (very rarely 1); Area VI = 1 bar; Areas VII–VIII = 32–46 in two rows. Area IV without bars. Paragnaths on oral ring tend to be larger than those on maxillary ring. Neurochaetae with homogomph spinigers and 1–3 robust heterogomph falcigers in supra-acicular fascicle; heterogomph falcigers and spinigers in subacicular fascicle (Hutchings et al. 1991).

##### Distribution and habitat.

The species was found in Indo-West Pacific, inhabiting intertidal rocky shores.

##### Remarks.

The specimens in the present study agree well with previous descriptions of Hutchings et al. (1991). Morphology of the reproductive stage of this species is similar to that of *Perinereis helleri*. Characteristic features include a single short bar-shaped paragnath on each Area VI.

While *P. nigropunctata* is one of the most abundant *wawo* worms, this species was not found among *nyale* worms. The non-reproductive stage specimens obtained in the present work complement the collection of this species at MZB.

#### Perinereis obfuscata

Taxon classificationAnimaliaPhyllodocidaNereididae

(Grube, 1878)

22074163-0F37-5414-9006-10360EF8C226

Fig. 9D–F

Nereis (Perinereis) obfuscata Grube, 1878: 86–87. Horst 1924: 173–174, pl. 34, figs 5, 6.
Nereis obfuscata . – Ehlers 1920: 51.
Perinereis cultrifera var. *obfuscata*. – Monro 1939: 398–399.
Perinereis obfuscata . – Monro 1931: 16–18, fig. 10a–d. Hutchings et al. 1991: 257–258, fig. 12a–c.

##### Type locality.

Philippines.

##### Material examined.

MZB. Pol. 00288 (2 specimens) and MZB. Pol. 00342 (1 epitokous specimen), collected by Joko Pamungkas on 19 December 2017 from Barrang Lompo Island: Barrang Lompo Village, Sangkarrang Island District, Makassar City, South Sulawesi Province (5°02'45.7"S, 119°19'43.4"E); MZB. Pol. 00289 (4 specimens), collected by Joko Pamungkas on 23 November 2017 from Warmar Island: Siwalima Village, Aru Islands District, Aru Islands Regency, Maluku Province (5°45'26.9"S, 134°13'23.5"E); MZB. Pol. 00291 (1 specimen), collected by Joko Pamungkas on 17 November 2017 from Biak Island: Yenusi Village, East Biak District, Biak Numfor Regency, Papua Province (1°10'32.0"S, 136°12'23.9"E).

##### Diagnosis.

Body color in alcohol cream with brown pigmentation on prostomium and three transverse patches on anterior dorsal chaetigers. Prostomium trapezoidal with shallow anterior median furrow. Palps with cylindrical palpophores and rounded palpostyles. Longest tentacular cirri extending to chaetiger 1. Peristomium relatively long. Pharynx with conical paragnaths on both rings, smooth bars present on Area VI, arranged as follows. Area I = 2–9; Area II = 7–27 in 23 crescent-shaped rows (usually between 10–20); Area III = 13–32, sometimes with one or two cones displaced laterally, separate from the main group; Area IV = 10–31; Area V = 1 (very rarely 2); Area VI = 1 short crescent-shaped bar; Areas VII–VIII = 22–38 in two rows. Area IV without bars. Neurochaetae with homogomph spinigers and heterogomph falcigers in supra-acicular fascicle; heterogomph falcigers and few heterogomph spinigers in subacicular fascicle (Hutchings et al. 1991).

##### Distribution and habitat.

The species was found in Australia, Indonesia, and Philippines, inhabiting intertidal sandy shores with reef rubble.

##### Remarks.

The specimens in the present study agree well with previous descriptions. These specimens represent the first record of this species in the MZB collection. Characteristic features are the prominent paragnaths of the oral ring, including the single short crescent-shaped bars on each Area VI.

#### Perinereis singaporiensis

Taxon classificationAnimaliaPhyllodocidaNereididae

(Grube, 1878)

FA3B57D3-BCCC-54E9-94FB-6DCE3B461A02

Fig. 10A, B

Nereis (Perinereis) singaporiensis Grube, 1878: 84–85. Horst 1924: 169–170, pl. XXXIV, figs 1, 2.
Perinereis singaporiensis . – Pruvot 1930: 55–56, pl. 3, figs 62–64. Fauvel 1932: 103. Wu 1967: 67–68. Hylleberg et al. 1986: 9–10, fig. 5A–K. Hutchings et al. 1991: 262–263, fig. 15a–e.

##### Type locality.

Singapore.

**Figure 10.**
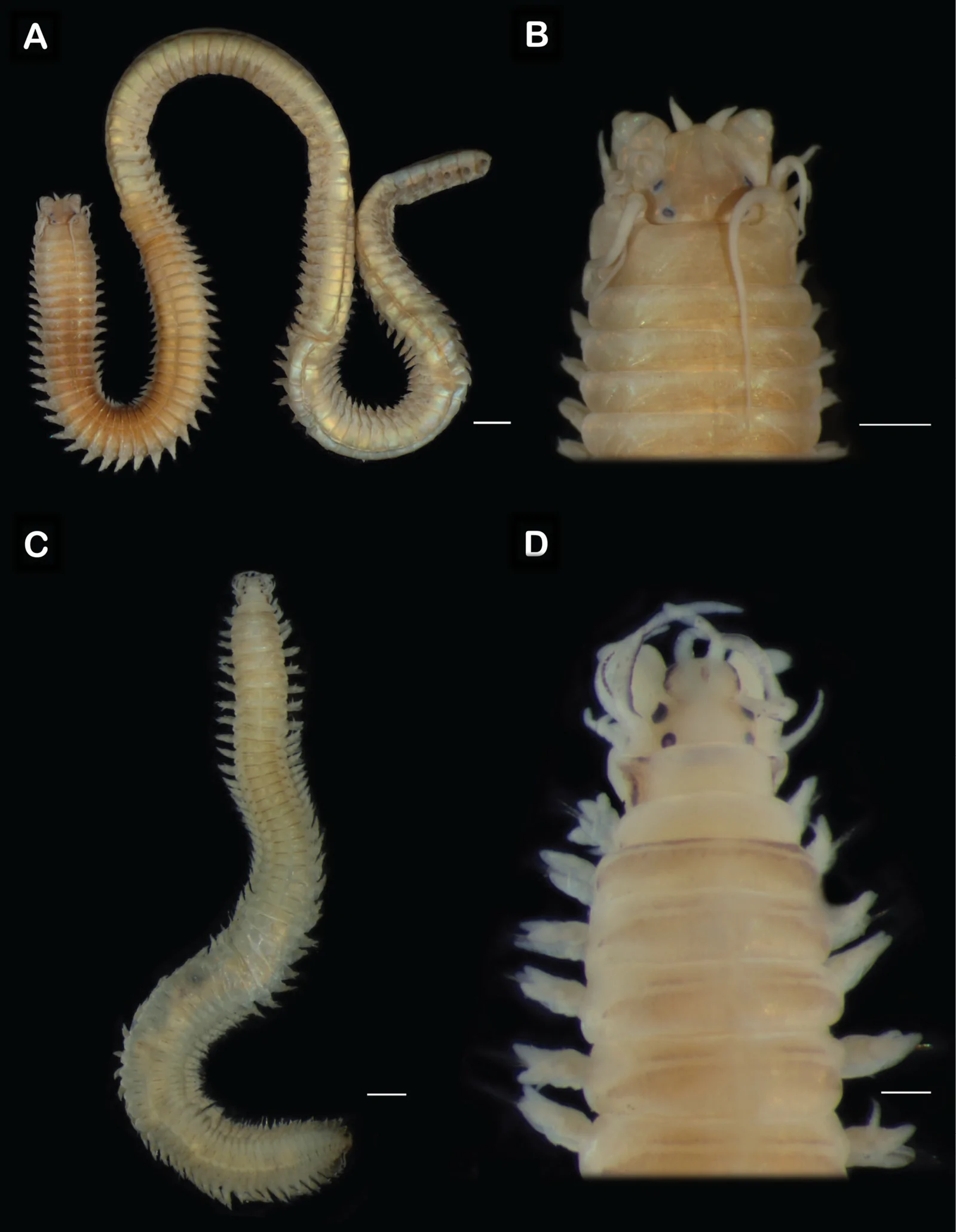
*Perinereis singaporiensis*. **A**. Whole body; **B**. Anterior end (dorsal view); **C**. *Perinereis* sp. (whole body); **D**. Anterior end (dorsal view). Scale bars: 2 mm (**A**); 1 mm (**B, C**); 0.25 mm (**D**).

##### Material examined.

MZB. Pol. 00287 (3 specimens), collected by Joko Pamungkas on 15 December 2017 from Lembeh Island: Mawali Urban Village, North Lembeh District, Bitung City, North Sulawesi Province (1°26'35.3"N, 125°13'48.9"E).

##### Diagnosis.

Body color in alcohol cream with brown pigmentation on dorsum of anterior and median chaetigers, fading posteriorly. Prostomium trapezoidal, elongate, rectangular anteriorly. Eyes with distinct lenses. Antennae triangular. Palps with small round palpostyles. Longest tentacular cirri extending to chaetiger 4. Peristomium relatively long. Pharynx with conical paragnaths on both rings, smooth bars present on Area VI, arranged as follows. Area I = 1–2; Area II = 7–21; Area III = 14–41 in a central patch with up to eight cones laterally placed, separate from this group; Area IV = 14–36; Area V = 3; Area VI = 2 (as paired bars); Area VII = 34–56 in two rows. Paragnaths on maxillary edge of oral ring smaller than those in oral or posterior position. Area IV without bars. Neurochaetae with homogomph spinigers and heterogomph falcigers in supra-acicular fascicle, and heterogomph spinigers as well as heterogomph falcigers in subacicular fascicle (Hutchings et al. 1991).

##### Distribution and habitat.

The species was found in Australia, India, Indonesia, New Caledonia, Philippines, Singapore, and Taiwan, inhabiting intertidal sandy shores with reef rubble.

##### Remarks.

The specimens in the present study agree well with previous descriptions and represent the first record of this species in the MZB collection. Characteristic features are the paired bar-shaped paragnaths on each Area VI.

#### Perinereis

Taxon classificationAnimaliaPhyllodocidaNereididae

sp.

F9400919-6828-5594-96E2-C33B409473F9

Fig. 10C, D

##### Material examined.

MZB. Pol. 00279 (2 specimens), collected by Joko Pamungkas on 24 November 2017 from Warmar Island: Wangel Village, Aru Islands District, Aru Islands Regency, Maluku Province (5°45'05.1"S, 134°11'43.2"E).

##### Diagnosis.

Specimens complete. Body color in alcohol cream with longitudinal narrow brown bands on two longest pairs of peristomial cirri, and transverse narrow brown bands on middle dorsal segments (one band on anterior margin of each segment) – transverse bands faint on anterior and posterior segments. Prostomium with entire anterior margin. Four circular black eyes present with anterior and posterior eyes arranged in an almost longitudinal row. Antennae conical. Palps biarticulated with subconical palpophores and rounded palpostyles. Peristomium width smaller than that of first chaetiger. Four pairs of smooth peristomial cirri with distinct cirrophores present; longest cirri reaching chaetiger 3. Paragnaths present on both rings. Area VI with short smooth bars. Chaetiger 1 more narrow than chaetiger 2. Notoaciculae of chaetigers 1 and 2 absent. Notochaetae without homogomph falcigers; neurochaetae with heterogomph falcigers in dorsal fascicles on anterior chaetigers.

##### Distribution and habitat.

The species was found in Maluku, Indonesia, inhabiting intertidal rocky shores.

##### Remarks.

Further identification is required to reveal the identity of the present *Perinereis* specimens (comparative material is ideally needed).

#### Platynereis

Taxon classificationAnimaliaPhyllodocidaNereididae

sp.

DF189CB1-09BB-5230-8F00-CF4893410279

Fig. 11A, B

##### Material examined.

MZB. Pol. 00280 (1 specimen), collected by Joko Pamungkas on 15 December 2017 from Lembeh Island: Mawali Urban Village, North Lembeh District, Bitung City, North Sulawesi Province (1°26'35.3"N, 125°13'48.9"E).

**Figure 11.**
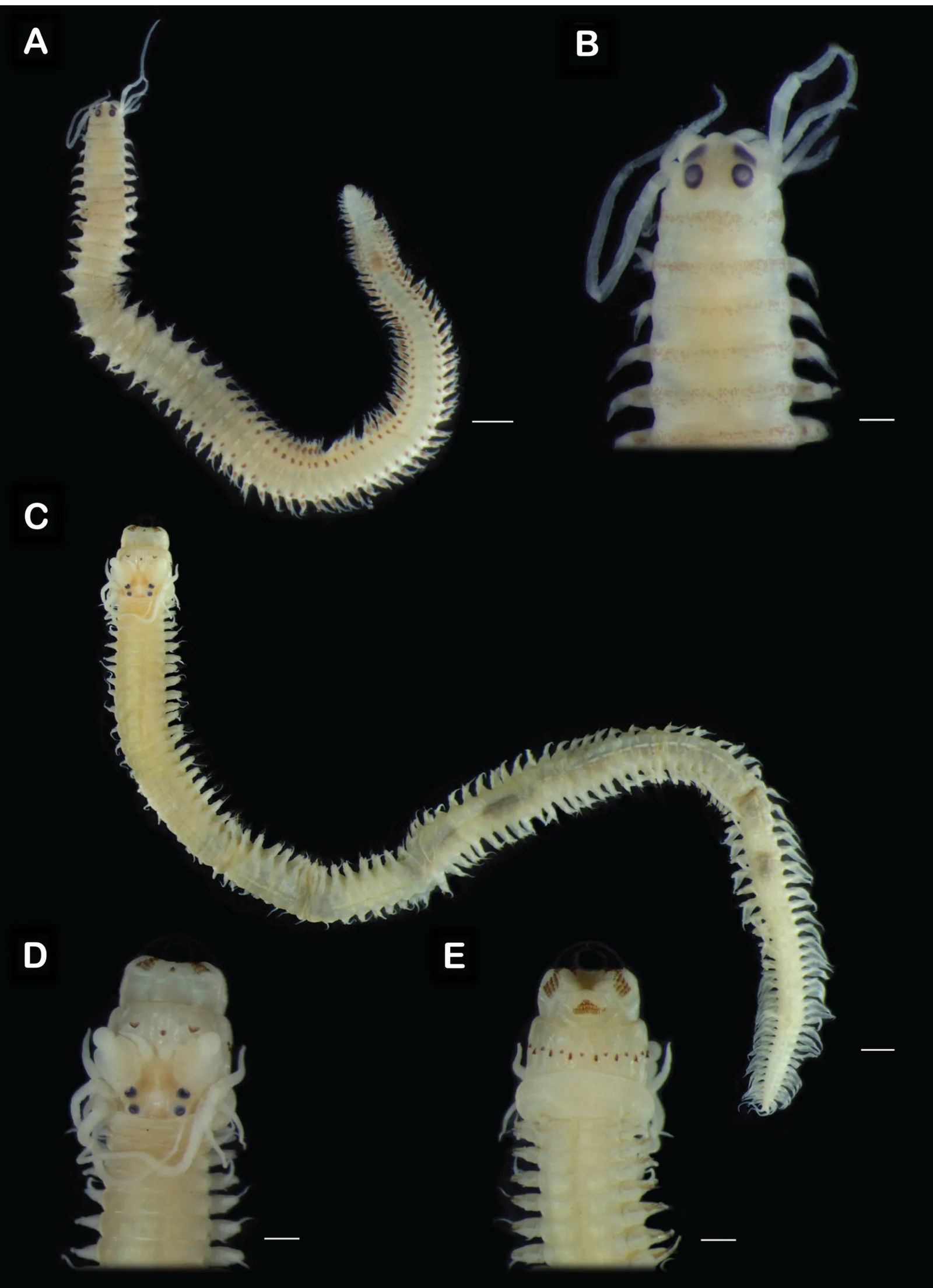
**A**. *Platynereis* sp. (whole body); **B**. Anterior end (dorsal view); **C**. *Pseudonereis trimaculata* (whole body); **D**. Anterior end (dorsal view); **E**. Anterior end (ventral view). Scale bars: 1 mm (**A, C**); 0.25 mm (**B**); 0.5 mm (**D, E**).

##### Diagnosis.

Single specimen complete. Body color in alcohol whitish cream with anterior dorsal segments having brown spots forming transverse lines (one line on each segment). Prostomium with entire anterior margin; length similar to width. Four black eyes present with anterior and posterior eyes arranged in a longitudinal row; anterior eyes semi-circle, ventrally directed; posterior eyes circular. Antennae conical, ventrally directed. Palps biarticulated with barrel-shaped palpophores and elongated palpostyles, ventrally directed. Peristomium length similar to that of chaetiger 1; middle and most lateral parts protruding anteriorly. Four pairs of peristomial cirri present with longest cirri reaching chaetiger 8. Pharynx with small rod-like paragnaths in tight rows.

##### Distribution and habitat.

The species was obtained from North Sulawesi, Indonesia, inhabiting intertidal sandy shore with reef rubbles.

##### Remarks.

The single specimen in the present study agrees well with the generic descriptions of *Platynereis*. More specimens are required for further identification, as the present specimen is transitioning to a reproductive form (eggs seen in the parapodia of the posterior body part). While two Indonesian *Platynereis* species have been identified, i.e., *Platynereis abnormis* (Horst, 1924) and *Platynereis cristatus* (Horst, 1924), the present specimen is the first MZB’s collection of the genus.

#### Pseudonereis trimaculata

Taxon classificationAnimaliaPhyllodocidaNereididae

(Horst, 1924)

C69DAA00-840D-513C-BB5A-4C9AF6DB14A1

Fig. 11C–E

Nereis (Pseudonereis) trimaculata Horst, 1924: 1.
Pseudonereis trimaculata (Horst, 1924): 187–188, pl. XXXVI, figs 8, 9. Bakken 2007: 166–168, fig. 13A–I. Hsueh 2021: 502–507, figs 8A–F, 9A–F, 10A–E, 11A–C, 12A–F, 13A–F, 14A–F.

##### Type locality.

Between Geser and Seram Islands, Maluku Province, Indonesia.

##### Material examined.

MZB. Pol. 00292 (1 specimen), collected by Joko Pamungkas on 23 January 2018 from Bali Island: Pemutih Beach, Pecatu Village, South Kuta District, Badung Regency, Bali Province (8°48'29.9"S, 115°06'27.3"E); MZB. Pol. 00293 (2 specimens), collected by Joko Pamungkas on 15 December 2017 from Lembeh Island: Mawali Urban Village, North Lembeh District, Bitung City, North Sulawesi Province (1°26'35.3"N, 125°13'48.9"E); MZB. Pol. 00295 (2 specimens), collected by Joko Pamungkas on 21 November 2017 from Ambon Island: Hutumuri Village, South Leitimur District, Ambon City, Maluku Province (3°41'46.1"S, 128°17'49.0"E); MZB. Pol. 00296 (9 specimens), collected by Joko Pamungkas on 27 January 2018 from Kalimantan Island: Prapatan Urban Village, South Balikpapan District, Balikpapan City, East Kalimantan Province (1°16'53.5"S, 116°48'39.3"E).

##### Diagnosis.

Prostomium with entire anterior margin, one pair of cirriform frontal antennae and two pairs of eyes. Maxillary ring of pharynx with paragnaths, arranged in discrete areas, Areas II–IV arranged in regular comb-like rows. Area I = 1–2 conical paragnaths, in a longitudinal row when 2; Area II = 23–38 p-bar paragnaths in 4 rows; Area III = 51–69 p-bar paragnaths in 4 rows; Area IV = 50–78 including p-bar paragnaths in 4 rows additional cones and 2–4 p-bars towards jaws. Areas V and VI present as distinct groups. Area V = 1–3 large conical paragnaths present, arranged in a triangular pattern when 3; Area VI = 1 shield-shaped bar present, cones absent; Areas VII–VIII = 20–24 large conical paragnaths of similar size and interspersed p-bars present in two alternating rows. Jaws with dentate cutting edge, brown, with five or six teeth. Tentacular belt length greater than that of chaetiger 1. Tentacular cirri with distinct cirrophores; longest cirri extending to chaetigers 5–7 (Bakken 2007).

##### Distribution and habitat.

The species was found in Indonesia, Western Australia, eastern and southern Taiwan, inhabiting intertidal rocky shores.

##### Remarks.

The specimens in the present study agree well with previous descriptions and are the first MZB’s collection of the species.

#### Solomononereis marauensis

Taxon classificationAnimaliaPhyllodocidaNereididae

Gibbs, 1971

A9AD5AE2-0E46-5250-A225-6C69DD4C6D11

Fig. 12

Solomononereis marauensis Gibbs, 1971: 152–153, fig. 8a–h. Hutchings and Reid 1991: 59–60. Pamungkas and Glasby 2015: 19, fig. 5D.

##### Type locality.

Marau Sound, Guadalcanal, Solomon Islands.

**Figure 12.**
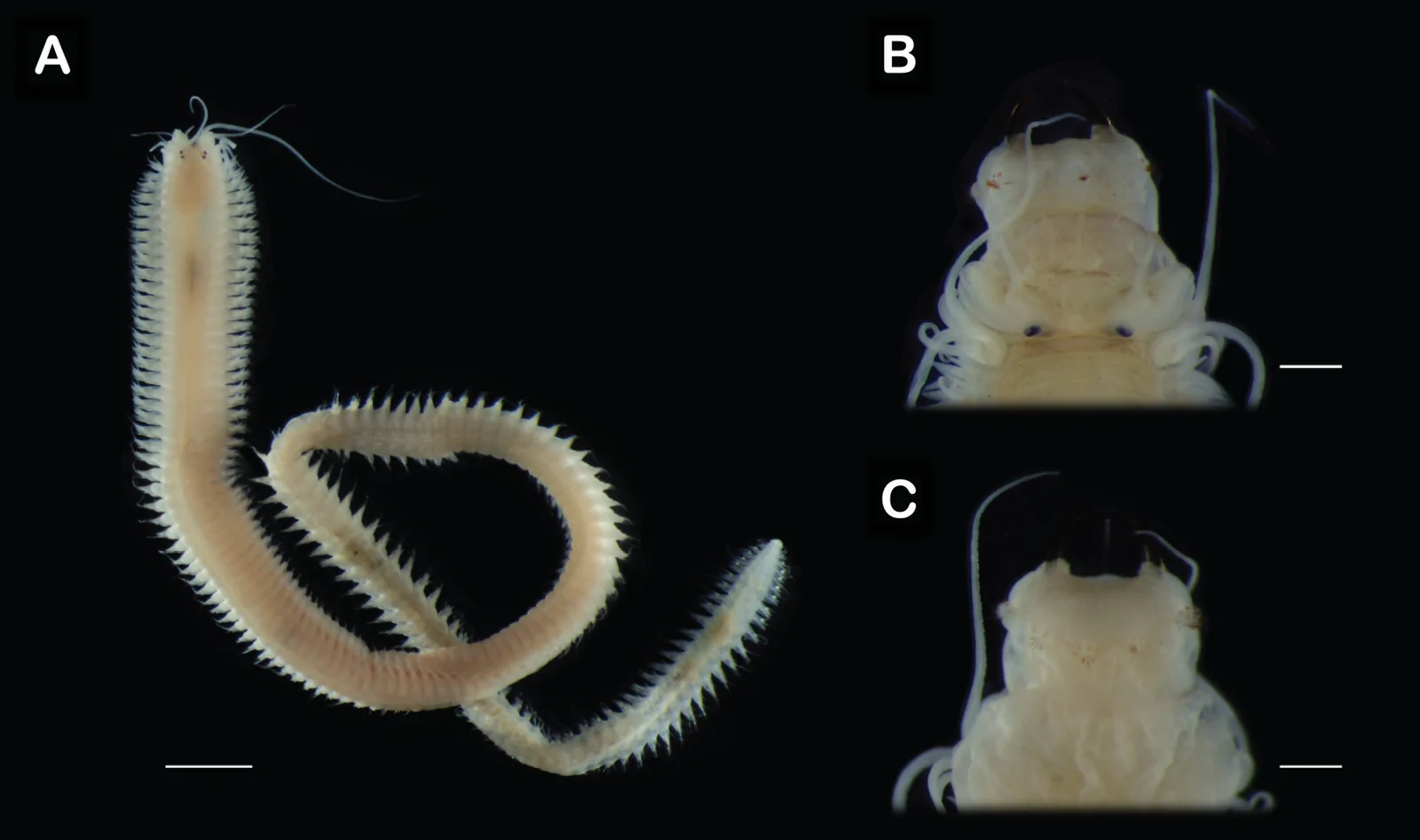
*Solomononereis marauensis*. **A**. Whole body; **B**. Anterior end with everted proboscis (dorsal view); **C**. Anterior end with everted proboscis (ventral view). Scale bars: 2 mm (**A**); 0.5 mm (**B, C**).

##### Material examined.

MZB. Pol. 00250 (1 specimen), collected by Joko Pamungkas on 7 October 2009 from Nusalaut Island: Titawaai Village, Nusalaut District, Central Maluku Regency, Maluku Province (3°40'46.8"S, 128°45'45.8"E); MZB. Pol. 00251 (2 specimens), collected by Joko Pamungkas on 3 September 2015 from Ambon Island: Waiheru Village, Teluk Ambon Baguala District, Ambon City, Maluku Province (3°38'02.1"S, 128°13'14.9"E); MZB. Pol. 00252 (3 specimens), collected by Joko Pamungkas on 4 September 2015 from Ambon Island: Poka Village, Teluk Ambon Baguala District, Ambon City, Maluku Province (3°39'17.0"S, 128°11'54.9"E).

##### Comparative material.

*Solomononereis marauensis*. MZB. Pol. 00176 (1 female), collected as heteronereids locally known as ‘*laor/wawo*’ by Robert Alik on 19 March 2014 from Ambon Island: Suli Village, Salahutu District, Central Maluku Regency, Maluku Province (3°37'38.2"S, 128°18'25.0"E).

##### Diagnosis.

Body color in alcohol pale brown to cream. Prostomium rectangular with antennae arising from extreme corners of distal margin. Palps globular with stout biarticulate palpophores and small palpostyles. Peristomium relatively narrow. All paragnaths rod-like, arranged in eight discrete groups on maxillary ring only as follows. Area I = 1–7; Area II = 5–12; Area III = 2–10 medially and 0–7 paragnaths laterally; Area IV = 3–14. Notopodia with homogomph spinigers throughout and homogomph falcigers in far posterior chaetigers. Neuropodia with homo- and sesquigomph spinigers as well as a single heterogomph falciger in supra-acicular fascicle, and heterogomph spinigers in subacicular fascicle (Hutchings and Reid 1991).

##### Distribution and habitat.

The species was found in Maluku (Indonesia), Marau Sound (Solomon Islands), and northern Australia, inhabiting intertidal sandy shores.

##### Remarks.

The specimens in the present study agree well with the descriptions of Hutchings and Reid (1991). *Solomononereis marauensis* is often misspelled as *Solomononereis merauensis* (e.g., Pamungkas and Glasby 2015). Pamungkas and Glasby (2015) found a few individuals of this species among *wawo* worms. The non-reproductive stage specimens obtained in the present work complement the collection of this species at MZB.

### Species discovery of Indonesian nereidids

From 1865 to 2025, 84 nereidid species, belonging to 24 genera reported in 29 publications, have been identified from Indonesian waters (Table 3). Of the 84 Indonesian nereidid species, 44 species (15 genera in 16 publications) were described as new to science. Almost half of these new species (21 species) were described by Horst (1918a, 1918b, 1919, 1924), mostly based on the material collected during the Siboga Expedition. Except for *Dendronereides cilacapensis* and *Dendronereis javaensis*, all the other nereidid species were described by overseas scientists with specimens deposited abroad, mostly at NBC in the Netherlands (Table 3).

**Table 3.** List of Indonesian nereidid species with their national geographic distribution and general habitat. Species names in bold were found in the present study.

**No**	**Species**	**Author**	**Locality/Location**	**Expedition/ Collector**	**Repository**	**Habitat**	**Reference**	**Note**
**Species described**								
1	*Ceratonereis dorsolineata*	(Horst, 1924)	Makassar Strait, off Spermonde Archipelago	SIBOGA (St. 75)	NBC	Offshore (pelagic)	Horst (1924)	-
2	***Composetia marmorata***	(Horst, 1924)	Seram Sea, off Geser Island, East Seram, Maluku; Ambon and Aru Islands, Maluku; Central Lombok, West Nusa Tenggara	SIBOGA (St. 172) and J. Pamungkas et al.	MZB, NBC, NTM and RCLA	Coastal (reef) and offshore (pelagic)	Horst (1924); Pamungkas (2015); Pamungkas and Glasby (2015); this study	*Nyale* and *wawo*; economic importance
3	*Ceratonereis ternatensis*	(Fischli, 1900)	Ternate, Maluku	W. Kükenthal	Gesellschaft Naturforschender Freunde zu Berlin?	Coastal?	Fischli (1903)	-
4	*Ceratonereis tripartita*	Horst, 1918b	Flores Sea, off Kawasang and Sailus Kecil Islands, South Sulawesi	SIBOGA (Sts 37 and 40)	NBC	Offshore (pelagic)	Horst (1918b, 1924)	-
5	Ceratonereis (Composetia) hyalognatha	(Ehlers, 1920)	Ambon, Maluku	Johannes Brock	ZMH	Coastal (intertidal)	Ehlers (1920)	-
6	*Dendronereides cilacapensis*	Pamungkas, Glasby, Wibowo & Tilic, 2025	Cilacap, Central Java	J. Pamungkas and E. S. Wibowo	MZB and SMF	Coastal (mangrove)	Pamungkas et al. (2025)	-
7	*Dendronereis javaensis*	Pamungkas, Glasby, Wibowo & Tilic, 2025	Brebes and Cilacap, Central Java	J. Pamungkas and E. S. Wibowo	MZB and SMF	Coastal (brackish water pond and mangrove)	Pamungkas et al. (2025)	Economic importance
8	Gnatholycastis brocki*	Ehlers, 1920	Ambon, Maluku	Johannes Brock	ZMB	Coastal (intertidal)	Ehlers (1920)	-
9	*Gymnonereis sibogae*	Horst, 1918b	Makassar Strait, off Spermonde Archipelago; Flores Sea, off Sailus Kecil and Selayar Islands, South Sulawesi	SIBOGA (Sts 37, 66 and 75)	NBC	Offshore (pelagic)	Horst (1918b); Pettibone (1970)	-
10	*Namalycastis meraukensis*	(Horst, 1918b)	Merauke, South Papua	J. W. R. Koch	NBC	Coastal (decayed wood of Sago-palm trees)	Horst (1918b)	-
11	*Namalycastis nipae*	(Pflugfelder, 1933)	Belawan, Medan, North Sumatra	LIMNOLOGISCHE SUNDA	ZMB?	Coastal (mangrove)	Pflugfelder (1933)	-
12	*Namalycastis rhodochorde*	Glasby, Miura, Nishi & Junardi, 2007	Sungai Kakap, Kubu Raya, West Kalimantan	Junardi	NTM	Coastal (mangrove)	Glasby et al. (2007)	Economic importance
13	*Namalycastis terrestris*	(Pflugfelder, 1933)	Belawan, Medan, North Sumatra	LIMNOLOGISCHE SUNDA	ZMB?	Non-marine (terrestrial)	Pflugfelder (1933)	-
14	*Namalycastis vivax*	(Pflugfelder, 1933)	Perbaungan, Serdang Bedagai, North Sumatra	LIMNOLOGISCHE SUNDA	ZMB?	Coastal (mangrove)	Pflugfelder (1933)	-
15	*Namanereis amboinensis*	(Pflugfelder, 1933)	Ambon, Maluku	LIMNOLOGISCHE SUNDA	ZMB?	Coastal (under dead leaf sheaths of coconut palms)	Pflugfelder (1933)	-
16	*Namanereis catarractarum*	(Feuerborn, 1931)	Lake Ranau, South Sumatra; Bedali, Malang, East Java	LIMNOLOGISCHE SUNDA	ZMB?	Non-marine (freshwater and terrestrial)	Feuerborn (1931); Glasby et al. (1990)	-
17	*Neanthes gisserana*	(Horst, 1924)	Seram Sea, off Geser Island, East Seram, Maluku	SIBOGA (St. 172)	NBC	Offshore (pelagic)	Horst (1924); Villalobos-Guerrero and Idris (2021)	-
18	*Neanthes indica*	(Kinberg, 1865)	Bangka Strait	EUGENIE	NRS	Offshore?	Kinberg (1865)	-
19	*Neanthes vitabunda*	(Pflugfelder, 1933)	Belawan, Medan, North Sumatra	LIMNOLOGISCHE SUNDA	ZMB?	Non-marine (terrestrial)	Pflugfelder (1933)	-
20	*Nereis abyssicola*	(Horst, 1924)	Halmahera Sea, off Kofiau Island, Raja Ampat, West Papua	SIBOGA (St. 161)	NBC	Offshore (deep sea at 798 m b.s.l.)	Horst (1924)	-
21	*Nereis baliensis*	(Horst, 1924)	Bali Sea, off Kangean Island	SIBOGA (St. 12)	NBC	Offshore (deep sea at 289 m b.s.l.)	Horst (1924)	-
22	*Nereis batjanensis*	(Horst, 1924)	Molucca Sea, off Bacan Island, North Maluku	SIBOGA (St. 140)	NBC	Offshore (pelagic)	Horst (1924)	-
23	*Nereis buitendijki*	Horst, 1919	Malacca Strait, off Sumatra Island	P. Buitendijk	NBC	Offshore (pelagic)	Horst (1919, 1924)	-
24	*Nereis heteromorpha*	(Horst, 1924)	Seram Sea, off Geser Island, East Seram, Maluku	SIBOGA (St. 172)	NBC	Offshore (pelagic)	Horst (1924)	-
25	*Nereis macropis*	Ehlers, 1920	Ambon, Maluku	Johannes Brock	N/A	Coastal (intertidal)	Ehlers (1920)	
26	*Nereis nouhuysi*	Horst, 1918a	Taliabu Island, North Maluku; Pijot, East Lombok, West Nusa Tenggara	J. W. van Nouhuys and SIBOGA (St. 33)	NBC	Coastal (pelagic in estuary)	Horst (1918a, 1924)	-
27	*Nereis onychophora*	Horst, 1918b	Seram Sea, off Geser Island, East Seram, Maluku	SIBOGA (St. 172)	NBC	Offshore (pelagic)	Horst (1918b, 1924)	-
28	*Nereis profundi*	Kirkegaard, 1956	Banda Sea, Maluku	GALATHEA	ZMUC	Offshore (deep sea at ~ 6000 m b.s.l.)	Kirkegaard (1956)	-
29	*Nereis quoyii*	Quatrefages, 1866	Seram Sea, Maluku	ASTROLABE and Quoy and Gaimard	N/A	Offshore?	Quatrefages (1866)	-
30	*Nereis sumbawaensis*	(Horst, 1924)	Saleh Bay, Sumbawa, West Nusa Tenggara	SIBOGA (St. 312)	NBC	Offshore (deep sea at 274 m b.s.l.)	Horst (1924)	-
31	*Nereis thysanota*	Ehlers, 1920	Damar Besar Island, Jakarta	Johannes Brock	ZMH	Coastal (coral reef)	Ehlers (1920)	-
32	*Nereis trifasciata vandersandei*	(Horst, 1924)	Savu Sea, off East Flores	G. A. J. van der Sande	NBC	Offshore (pelagic)	Horst (1924)	-
33	*Nereis tydemani*	(Horst, 1924)	Seram Sea, off Geser Island, East Seram, Maluku	SIBOGA (St. 172)	NBC	Offshore (pelagic)	Horst (1924)	-
34	*Paraleonnates tenuipalpa*	(Pflugfelder, 1933)	Belawan, Medan, North Sumatra	LIMNOLOGISCHE SUNDA	ZMB?	Non-marine (terrestrial)	Pflugfelder (1933)	-
35	*Perinereis binongkae*	(Horst, 1924)	Binongko Island, Wakatobi, South East Sulawesi	SIBOGA (St. 220)	NBC	Offshore (pelagic)	Horst (1924)	-
36	*Perinereis caeruleis*	(Hoagland, 1920)	Talisei Island, North Sulawesi	ALBATROSS	USNM	Coastal	Hoagland (1920)	-
37	*Perinereis cavifrons*	Ehlers, 1920	Ambon, Maluku	Johannes Brock	ZMB	Coastal (intertidal)	Ehlers (1920)	-
38	*Perinereis dongalae*	(Horst, 1924)	Makassar Strait, off Dongala, Central Sulawesi	SIBOGA (St. 86)	NBC	Offshore	Horst (1924)	-
39	***Perinereis larentukana***	(Grube, 1881)	Larantuka, East Flores, East Nusa Tenggara; Nusalaut and Saparua, Maluku; Southwest Sumba, East Nusa Tenggara; Central Lombok, West Nusa Tenggara	Martens and J. Pamungkas et al.	MZB, SMF and ZMB	Coastal (sandy beach with reef)	Grube (1881); this study	*Nyale*; economic importance
40	*Perinereis tobeloana*	(Augener, 1933)	Tobelo, North Halmahera, North Maluku	N/A	ZMH	Coastal (reef)?	Augener (1933)	-
41	*Platynereis abnormis*	(Horst, 1924)	Banda Sea, South East Sulawesi	SIBOGA (St. 205)	NBC	Offshore (pelagic)	Horst (1924)	-
42	*Platynereis cristatus*	(Horst, 1924)	Banda Sea, off Kur Island, Maluku	SIBOGA (St. 250)	NBC	Offshore (pelagic)	Horst (1924)	-
43	***Pseudonereis trimaculata***	(Horst, 1924)	Seram Sea, off Geser Island, East Seram, Maluku; Badung, Bali; Bitung, North Sulawesi; Ambon, Maluku; Balikpapan, East Kalimantan	SIBOGA (St. 172) and J. Pamungkas et al.	MZB and NBC	Coastal (reef)	Horst (1924); this study	-
44	*Tylorrhynchus heterochetus*	(Quatrefages, 1866)	Java	N/A	N/A	N/A	Quatrefages (1866)	-
**Species identified**								
45	*Alitta succinea*	(Leuckart, 1847)	Belawan, Medan, North Sumatra	LIMNOLOGISCHE SUNDA	ZMB?	Non-marine (terrestrial)	Pflugfelder (1933)	-
46	*Ceratocephale* sp.	-	Natuna Islands, Riau Islands	ANAMBAS (St. NT02B)	MZB	Offshore	Al-Hakim and Glasby (2004)	-
47	*Ceratonereis australis*	Hartmann-Schröder, 1985	Ambon and Banda Sea, Maluku	J. Pamungkas et al.	MZB, NTM and RCLA	Coastal (pelagic)	Pamungkas (2015); Pamungkas and Glasby (2015)	*Wawo*; economic importance
48	Ceratonereis cf. perkinsi	Hartmann-Schröder, 1985	Ambon, Maluku	J. M. Martens et al.	ZMH	Coastal (pelagic)	Martens et al. (1995)	*Wawo*; economic importance
49	*Ceratonereis tentaculata*	Kinberg, 1865	Kawasang Island, South Sulawesi; Salebabu Island, North Sulawesi; Halmahera Sea, off Salomakie/Damar Island, North Maluku; Banda Sea, off Kur Island, Maluku; Timor Sea, Rote Island, East Nusa Tenggara; Savu Sea, off Sumbawa, West Nusa Tenggara	SIBOGA (Sts 40, 43, 66, 133, 144, 240, 250, 299, 310)	NBC	Offshore (mostly pelagic)	Horst (1924)	-
50	***Cheilonereis* sp**.	-	Warmar Island, Maluku	J. Pamungkas et al.	MZB	Coastal (reef)	This study	Possibly new species
51	Dendronereis pinnaticirris**	Grube, 1874	Surabaya and Madura, East Java	T. G. Pillai	NHMUK?	Coastal (brackish water ponds)	Pillai (1965)	-
52	*Gymnonereis phuketensis*	Hylleberg & Nateewathana, 1988	Natuna, Riau Islands	ANAMBAS (Sts NT06A, NB13B and NB19B)	MZB, NTM and ZRC	Offshore	Al-Hakim and Glasby (2004)	-
53	***Hediste* sp**.	-	Warmar Island, Maluku	J. Pamungkas et al.	MZB	Coastal (reef)	This study	Possibly new species
54	*Leonnates indicus*	Kinberg, 1865	Arafura, Flores, and Seram Seas; Selayar Island, South Sulawesi; Strait of Makassar	SIBOGA (Sts 164, 213, 273, 315) and van Kampen	NBC	Coastal (reef) and offshore	Qiu and Qian (2000)	-
55	*Leonnates persicus*	Wesenberg-Lund, 1949	Natuna Islands, Riau Islands	ANAMBAS (Sts NT05A, NT08A, NB15A and NB17B)	MZB, NTM and ZRC	Offshore	Al-Hakim and Glasby (2004)	-
56	*Namalycastis hawaiiensis*	(Johnson, 1903)	Bogor, West Java; Lake Ranau, South Sumatra	LIMNOLOGISCHE SUNDA	ZMB?	Non-marine (freshwater pond and lake)	Feuerborn (1931)	-
57	*Neanthes bongcoi*	Pillai, 1965	Sunda Strait; Indian Ocean, off West Java	SJADES (Sts CP02 and CP56)	LKCNHM?	Offshore (deep sea at 183–281 m b.s.l.)	Chuar et al. (2021)	-
58	**Neanthes cf. gisserana**	(Horst, 1924)	Ambon, Maluku; Central Lombok, West Nusa Tenggara	J. Pamungkas et al.	MZB, NTM, RCLA and SMF	Coastal (pelagic)	Pamungkas (2015); Pamungkas and Glasby (2015); this study	*Nyale* and *wawo* (possibly new species); economic importance
59	Neanthes cf. masalacensis	(Grube, 1878)	Ambon, Maluku	J. Pamungkas et al.	MZB, NTM and RCLA	Coastal (pelagic)	Pamungkas (2015); Pamungkas and Glasby (2015)	*Wawo* (possibly new species); economic importance
60	*Neanthes cricognatha*	(Ehlers, 1904)	Seram Sea, off Salawati Island, Raja Ampat, Southwest Papua	SIBOGA (St. 164)	NBC	Offshore	Horst (1924)	-
61	*Neanthes negomboensis*	De Silva, 1965	Surabaya, East Java	T. G. Pillai	NHMUK?	Coastal (brackish water pond)	Pillai (1965)	-
62	*Neanthes pachychaeta*	(Fauvel, 1918)	Kupang, East Nusa Tenggara; Ambon, Maluku	SIBOGA (St. 296)	NBC	Offshore (pelagic)	Horst (1919, 1924); Pamungkas and Glasby (2015)	*Wawo*; economic importance
63	***Neanthes unifasciata***	(Willey, 1905)	Ambon and Aru Islands, Maluku; Flores Sea, off Kawasang and Selayar Islands, South Sulawesi; Central Lombok, West Nusa Tenggara	J. M. Martens, Merton, J. Pamungkas et al. and SIBOGA (Sts 40 and 66)	MZB, NTM, RCLA, SMF and ZMH	Coastal (pelagic)	Ehlers (1918); Horst (1924); Martens et al. (1995); Pamungkas (2015); Pamungkas and Glasby (2015); this study	*Nyale* and *wawo*; economic importance
64	*Nectoneanthes* sp.	-	Natuna Islands, Riau Islands	ANAMBAS (St. NT04A)	MZB	Offshore	Al-Hakim and Glasby (2004)	-
65	Nereis cf. grayi	Pettibone, 1956	Indian Ocean, off West Java	SJADES (St. CP26)	LKCNHM?	Offshore (deep sea at 517–727 m b.s.l.)	Chuar et al. (2021)	-
66	*Nereis jacksoni*	Kinberg, 1865	Onrust Island, Jakarta	L. van Lummel	MZB	Coastal (reef)	Augener (1932)	-
67	*Nereis nigripes*	Ehlers, 1868	Makassar, South Sulawesi	SIBOGA (St. 71)	NBC	Coastal?	Horst (1924)	-
68	*Nereis* sp_Ambon_NTMW19037	-	Ambon, Maluku	J. Pamungkas et al.	MZB, NTM and RCLA	Coastal (reef and pelagic)	Pamungkas and Glasby (2015)	*Wawo* (new species candidate); economic importance
69	*Nicon pettiboneae*	de León-González & Salazar-Vallejo, 2003	Indian Ocean, off Central and West Java	SJADES (Sts CP37 and CP56)	LKCNHM?	Offshore (deep sea at 163–255 m b.s.l.)	Chuar et al. (2021)	-
70	*Perinereis aibuhitensis*	(Grube, 1878)	Makassar, South Sulawesi; Jakarta	P. N. van Kampen and Max Weber	NBC?	Coastal (marine fish pond and intertidal)	Horst (1924)	Economic importance
71	*Perinereis floridana*	(Ehlers, 1868)	Flores Sea, off Kawasang Island, South Sulawesi	SIBOGA (St. 40)	NBC	Offshore (pelagic)	Horst (1924)	-
72	Perinereis helleri***	(Grube, 1878)	Ambon and Aru Islands, Maluku; Bitung, North Sulawesi; Makassar, South Sulawesi; Southwest Sumba, East Nusa Tenggara	SIBOGA (St. 181), J. M. Martens et al. and J. Pamungkas et al.	MZB, NTM, NBC, RCLA, SMF and ZMH	Coastal (reef and pelagic)	Horst (1924); Martens et al. (1995); Pamungkas (2015); Pamungkas and Glasby (2015); this study	*Nyale* and *wawo*; economic importance
73	***Perinereis nigropunctata***	(Horst, 1889)	Ambon and Aru Islands, Maluku; East Lombok, West Nusa Tenggara; Makassar, South Sulawesi	SIBOGA (St. 34), J. M. Martens et al. and J. Pamungkas et al.	MZB, NTM, NBC, RCLA and ZMH	Coastal (reef and pelagic)	Horst (1924); Martens et al. (1995); Pamungkas (2015); Pamungkas and Glasby (2015); this study	*Wawo*; economic importance
74	*Perinereis nuntia*	Lamarck, 1818	Labuhan Pandan, East Lombok, West Nusa Tenggara; Aru Islands, Maluku; Pari Island, Jakarta	SIBOGA (Sts 34 and 272) and I. Al-Hakim	NBC and NTM	Coastal and offshore	Horst (1919, 1924); Glasby and Hsieh (2006)	Economic importance
75	***Perinereis obfuscata***	(Grube, 1878)	Bali Sea, off Kangean Island; Savu Sea, Sumba, East Nusa Tenggara; Makassar, South Sulawesi; Aru Islands, Maluku; Biak Numfor, Papua	SIBOGA (Sts 16 and 53) and J. Pamungkas et al.	MZB and NBC	Coastal (reef) and offshore	Horst (1924); this study	-
76	*Perinereis perspicillata*	(Grube, 1878)	Onrust Island, Jakarta	L. van Lummel	MZB	Coastal (reef)	Augener (1932)	-
77	***Perinereis singaporiensis***	(Grube, 1878)	Kwandang, North Gorontalo; Bitung, North Sulawesi	SIBOGA (St. 115) and J. Pamungkas et al.	MZB and NBC	Coastal (reef)	Horst (1924); this study	-
78	*Platynereis bengalensis*	(Willey, 1905)	Jedan Island, Aru Islands, Maluku	SIBOGA (St. 273)	NBC?	Offshore	Horst (1924)	-
79	Platynereis cf. australis	(Schmarda, 1861)	Flores Sea, Selayar Island, South Sulawesi	SIBOGA (St. 213)	NBC?	Coastal (pelagic in reef)	Horst (1924)	-
80	*Platynereis dumerilii*	(Audouin & Milne Edwards, 1833)	Seram Sea, Daram Island, Raja Ampat, West Papua; Timor Sea, Kupang, East Nusa Tenggara	SIBOGA (Sts 165 and 296)	NBC?	Offshore (pelagic)	Horst (1924)	-
81	*Platynereis polyscalma*	Chamberlin, 1919	Seram Sea, off Geser Island, East Seram, Maluku; Dobo, Aru Islands, Maluku	SIBOGA (Sts 172 and 272)	NBC?	Offshore (pelagic)	Horst (1924)	-
82	*Pseudonereis anomala*	Gravier, 1899	Flores Sea, Selayar Island, South Sulawesi	SIBOGA (St. 213)	NBC?	Coastal (reef)	Horst (1924)	-
83	***Solomononereis marauensis***	Gibbs, 1971	Ambon and Nusalaut, Maluku	J. Pamungkas et al.	MZB and NTM	Coastal (mangrove and pelagic in reef)	Pamungkas (2015); Pamungkas and Glasby (2015); this study	*Wawo*; economic importance
84	*Websterinereis foli*	(Fauvel, 1930)	Ambon, Maluku	J. M. Martens et al.	ZMH	Coastal (pelagic in reef)	Martens et al. (1995); Pamungkas and Glasby (2015)	*Wawo*; economic importance

* *Gnatholycastis brocki* is possibly a *Perinereis* species. Examination of the type specimens are required to confirm this. ** *Dendronereis pinnaticirris* may be *Dendronereis javaensis* as indicated by Pamungkas et al. (2025). *** *Perinereis cultrifera* identified by Martens et al. (1995) was considered to be *Perinereis helleri* as indicated by Pamungkas and Glasby (2015). Also, based on the morphological comparison conducted by JP, the European species *P. cultrifera* looks different from *P. helleri* despite the similarity in the paragnath arrangement.

**Figure 13.**
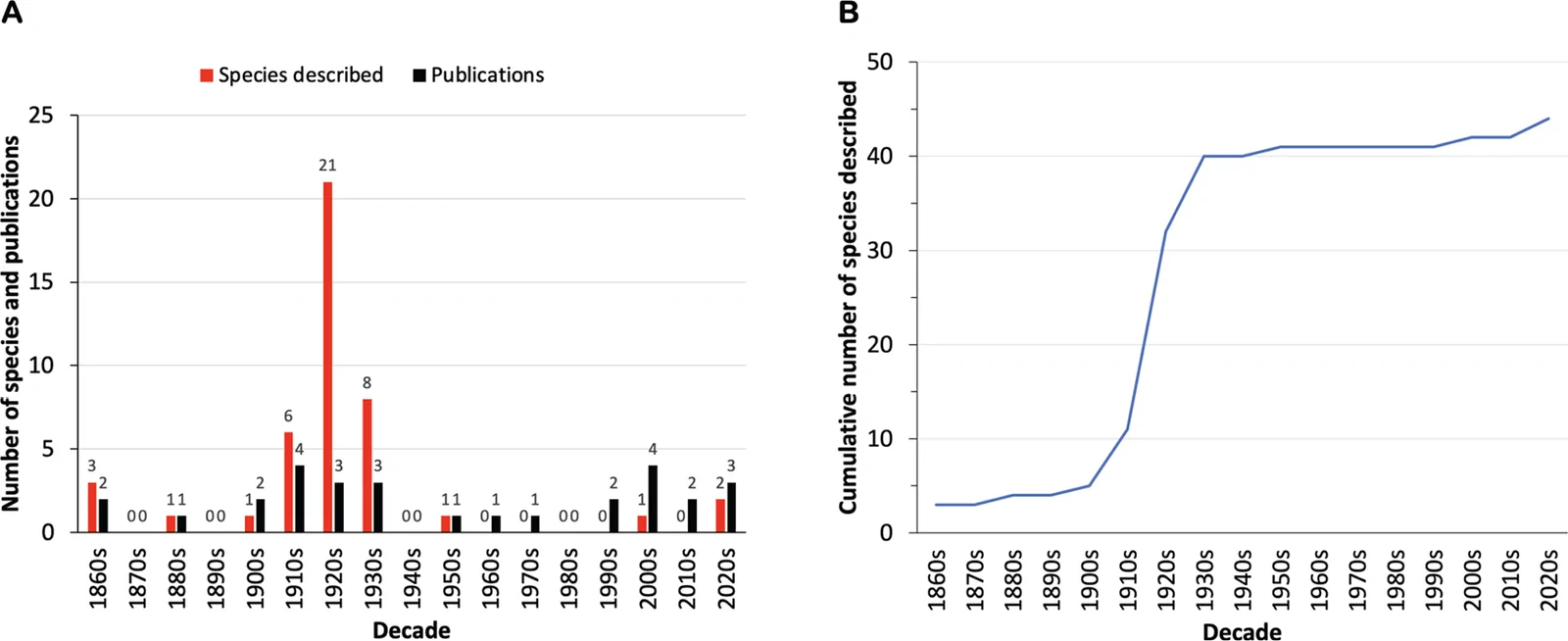
**A**. Number of nereidid species described and publications over time; **B**. Cumulative number of nereidid species described over time.

The first nereidid species described from Indonesia was *Neanthes indica* by Kinberg (1865) – the species seems to be endemic to the country. Thereafter, the number of species described peaked in the 1920s with 21 species (Fig. 13A). In the 1930s, the number of species described decreased to 8 species (the material was collected during the Limnologische Sunda Expedition). It then dropped sharply to 0–2 species for almost a century (Fig. 13A), resulting in the curve of the cumulative number of species described almost levelling off (Fig. 13B).

**Figure 14.**
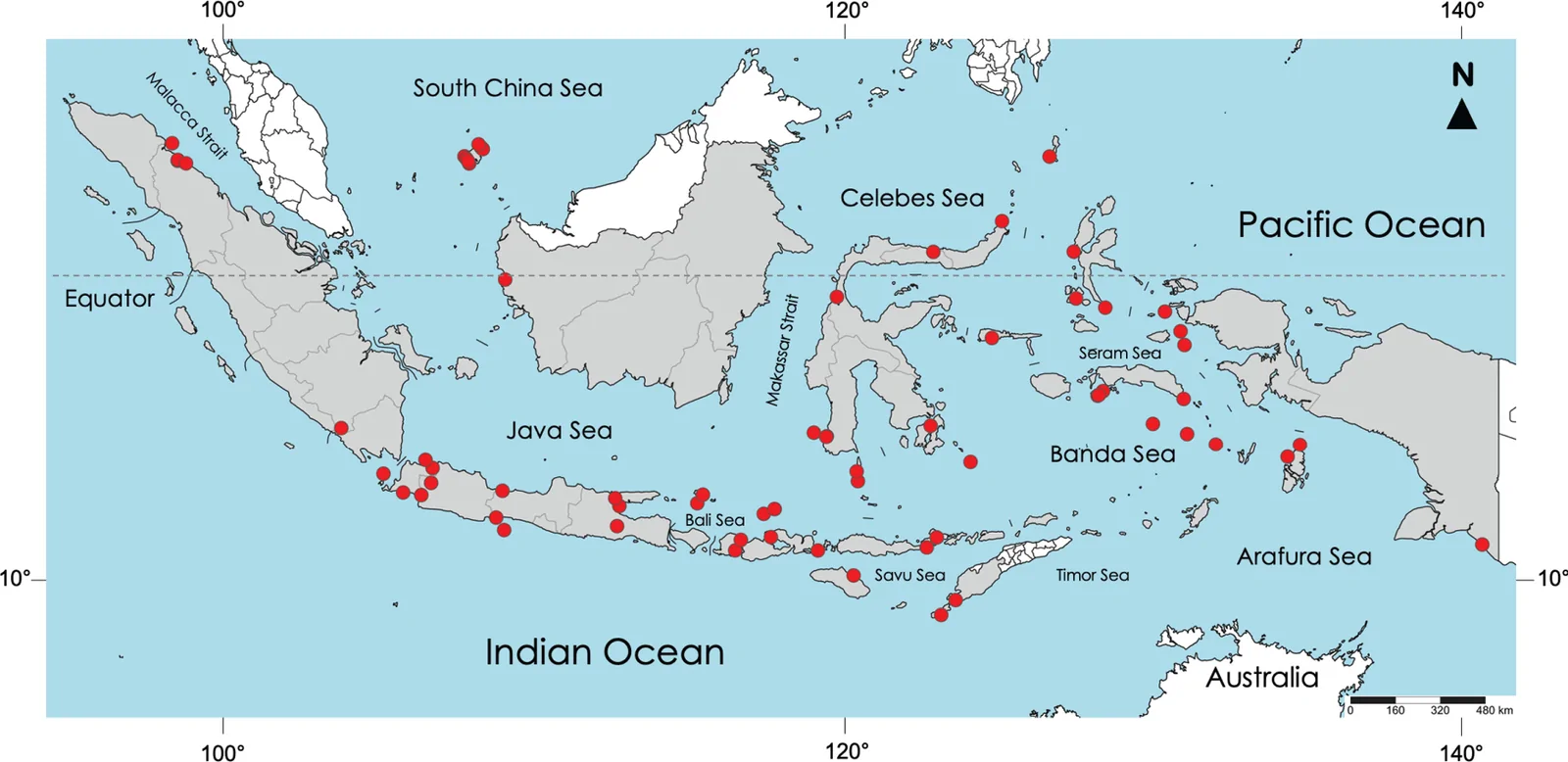
Map of nereidid distribution in Indonesia. Red dots indicate nereidid records obtained from taxonomic publications.

Of the 84 Indonesian nereidid species, 78 were marine, whereas six were non-marine. Most marine species were documented offshore (i.e., in shallow water areas) and in coastal areas, whereas seven species were obtained from deep-sea environments (Table 3, Fig. 14). In general, most sampling efforts were concentrated in the Coral Triangle, representing the stations of the Siboga Expedition. Sumatra, Kalimantan, and Papua are the three big islands with the least nereidid data (Fig. 14).

Both *nyale* and *wawo* hold economic importance as the worms are traded in traditional markets. Other nereidid species with economic importance include *D. javaensis*, *N. rhodochorde*, *P. aibuhitensis*, and *P. nuntia* species group. These species are caught and traded by locals for use as fishing baits (JP, pers. obs.). The *Perinereis nuntia* species group, in particular, is also cultivated for use as a natural feed for shrimp broodstock in some commercial companies (Asri T. Yuniar, pers. comm.).

### Key to Indonesian Nereididae genera

**Table:** 

1	Parapodia highly reduced (notopodia lacking distinct flattened lobes); pharynx completely lacking paragnaths or papillae; mostly freshwater or estuarine forms (subfamily Namanereidinae)	**2**
–	Parapodia well-developed (biramous, with distinct notopodial and neuropodial lobes); pharynx variable, often with paragnaths and/or papillae; mostly marine or estuarine forms	**3**
2	Three or four pairs of tentacular cirri; posterior dorsal cirri normal, not structurally expanded	*** Namanereis ***
–	Four pairs of tentacular cirri; posterior dorsal cirri elongated, flattened, and leaf-like	*** Namalycastis ***
3	Branchiae (gills) present as complex, branched structures arising from parapodia or dorsal cirri on mid-anterior segments	**4**
–	Branchiae absent (expanded/elongated lobes may be present, but not complexly branched or arborescent)	**5**
4	Branchiae developed as arborescent tufts arising directly from dorsal cirri	*** Dendronereis ***
–	Branchiae developed as modified, subdivided lobes of the dorsal notopodial ligules	*** Dendronereides ***
5	Eversible pharynx entirely smooth, completely lacking both paragnaths and soft papillae	*** Nicon ***
–	Eversible pharynx with some form of ornamentation (paragnaths, papillae, or both)	**6**
6	Transverse dorsal lamellae present commencing chaetigers 4–10	*** Ceratocephale ***
–	Transverse dorsal lamellae absent	**7**
7	Double ventral cirri in anterior segments; prostomium deeply indented anteriorly; pharynx with soft papillae but completely lacking chitinous paragnaths	*** Gymnonereis ***
–	Double ventral cirri absent; prostomium margin entire or only weakly indented; pharynx ornamentation variable	**8**
8	Chitinous paragnaths absent; pharynx completely bare or with very indistinct, sparse soft papillae	*** Websterinereis ***
–	Chitinous paragnaths present on at least one ring of pharynx	**9**
9	Dorsal cirrus terminally attached throughout; dorsal notopodial ligule absent, resembling a cirrophore of dorsal cirrus	*** Tylorrhynchus ***
–	Dorsal cirrus attachment variable; dorsal notopodial ligule usually present	**10**
10	Paragnaths arranged in distinct, comb-like pectinate rows on pharynx	*** Platynereis ***
–	Paragnaths not arranged in regular comb-like pectinate rows	**11**
11	Pharynx possessing specialized p-bars or shield-shaped paragnaths alongside conical ones	*** Pseudonereis ***
–	Pharynx lacking p-bars and shield-shaped paragnaths	**12**
12	Paragnaths exceptionally tiny, numerous, and uniformly rod-like across all 8 pharyngeal areas	*** Solomononereis ***
–	Paragnaths diverse, primarily containing standard conical denticles	**13**
13	Anterior margin of the prostomium clearly indented or cleft between antennae	*** Ceratonereis ***
–	Anterior margin of the prostomium entire (may be slightly indented, but not deeply cleft)	**14**
14	Maxillary ring with paragnaths; oral ring completely bare of paragnaths	*** Composetia ***
–	Both pharyngeal rings possessing some arrangement of chitinous paragnaths	**15**
15	Maxillary ring bearing a mix of both chitinous paragnaths and soft papillae	*** Leonnates ***
–	Maxillary ring bearing only chitinous paragnaths	**16**
16	Oral ring bearing soft papillae while the maxillary ring has only paragnaths	*** Paraleonnates ***
–	Both rings bearing exclusively chitinous paragnaths	**17**
17	Area VI of the oral ring characterized by one or more distinct, transverse smooth bars	*** Perinereis ***
–	Area VI of the oral ring lacking transverse bars, possessing only conical or pyramidal paragnaths	**18**
18	Upper margin of the posterior dorsal ligules extremely broad, strap-like, or leaf-like, bearing the dorsal cirrus at distal tip	*** Alitta ***
–	Posterior dorsal ligules expanded or not; if upper margin of dorsal ligule expanded, dorsal cirrus is attached sub-distally	**19**
19	Peristomium highly modified ventrally into a collar-like structure flaring forward around the head	*** Cheilonereis ***
–	Peristomium, ventrally smooth, lacking an enlarged overlapping head-flap	**20**
20	Oral ring paragnaths highly numerous, coalescing into broad, nearly continuous bands	*** Nectoneanthes ***
–	Oral ring paragnaths distinct, grouped into clearly isolated Areas (V–VIII)	**21**
21	Posterior notopodia possessing distinct, curved homogomph falcigers	*** Nereis ***
–	Posterior notopodia completely lacking homogomph falcigers	**22**
22	Notopodial prechaetal lobes usually well-developed and distinct throughout the entire body length; specialized simple chaetae in the neuropodia usually absent	*** Neanthes ***
–	Notopodial prechaetal lobes reduced, missing, or fused to adjacent structures; posterior segments bear specialized simple chaetae in the neuropodia formed by the fusion of the blade and shaft	*** Hediste ***

## Discussion

Nereididae is a species-rich polychaete family and ranks third after Syllidae and Polynoidae in terms of the number of species described (Pamungkas et al. 2019). In Indonesia, Nereididae was reported to be the polychaete family with the most species (Pamungkas and Glasby 2019).

We found that over the period of ca 1.5 centuries (1865–2025), 84 nereidid species (24 genera in 29 publications) have been identified from Indonesian waters. The number of nereidid species identified in the present study is slightly higher than that reported by Pamungkas and Glasby (2019), i.e., 75 species, mainly due to data addition (mainly addition of new species and records, as well as previously missing records) and correction (mainly removal of non-Indonesian species and unaccepted species names) – see Appendix 1. Of these 84 species, 44 species (15 genera in 16 publications) were new to science. This number is disproportionately small – i.e., ~ 6% – compared to the global number of nereidid species. Contrary to its status as a country with mega-marine biodiversity, the contribution of Indonesia to global nereidid species discovery has therefore been insignificant. As can be seen in Fig. 13B, the curve of the cumulative number of nereidid species described seems to be reaching an asymptote since the 1930s, not because most conspicuous species have already been described, but rather due to the taxonomic impediment faced by the country (Siallagan et al. 2023; Idris et al. 2025). Primarily, Indonesia lacks polychaete taxonomists, which results in a limited number of taxonomic investigations on the taxon (past species discoveries largely depended on the work of overseas scientists).

In the present study, five nereidid species of *nyale* were identified from East and West Nusa Tenggara (two sampling sites on two occasions in total). This number is much lower than that of *wawo* from Ambon, i.e., 13 nereidid species (Pamungkas and Glasby 2015), mainly due to unequal sampling efforts (the *wawo* worms were collected from six sampling sites on 11 occasions in total). *Wawo* worms are in fact more well studied than *nyale* and have also been previously investigated by Martens et al. (1995). Despite the species richness difference, our study reveals the fact that nereidid *nyale* and *wawo* are mostly of the same species composition, inhabiting the same habitat, i.e., coastal waters with reef and sand. Table 3 shows that *nyale* and *wawo* species also occur in several areas where locals do not have a tradition of consuming the worms. These findings therefore refute the common belief that *nyale* and *wawo* are unique to Nusa Tenggara and Maluku, respectively, and raise the possibility that the poorly documented *meci* of Timor-Leste also comprises a similar nereidid species composition. This question will be addressed in a separate study by CJG and JP.

## Conclusions

In the present study, 20 nereidid species in 10 genera collected from the Indonesian part of the Coral Triangle have been identified, with *Perinereis* and *Neanthes* being the genera with the most species. Two genera, i.e., *Cheilonereis* and *Hediste*, represent the first records for Indonesian waters, and three species, i.e., *Cheilonereis* sp., *Hediste* sp. and Neanthes cf. gisserana, are potentially new to science. Five *nyale* species have also been identified, i.e., *Composetia marmorata* (Horst, 1924), Neanthes cf. gisserana, *Neanthes unifasciata* (Willey, 1905), *P. helleri*, and *P. larentukana*; these findings indicate that *nyale* are mostly the same species as *wawo*.

We found that 84 Indonesian nereidid species (24 genera in 29 publications) have been identified between 1865 and 2025, 44 of which are new to science. These data shows that Indonesia’s contribution to the global nereidid species discovery has been disproportionately small. Further taxonomic investigations involving integrative studies are, therefore, required and should be focused on the three big islands with the least data, i.e., Sumatra, Kalimantan, and Papua.

## Supplementary Material

XML Treatment for Alitta

XML Treatment for Ceratonereis

XML Treatment for Ceratonereis

XML Treatment for Cheilonereis

XML Treatment for Composetia marmorata

XML Treatment for Hediste

XML Treatment for Neanthes cf. gisserana

XML Treatment for Neanthes unifasciata

XML Treatment for Neanthes

XML Treatment for Neanthes

XML Treatment for Neanthes

XML Treatment for Perinereis helleri

XML Treatment for Perinereis larentukana

XML Treatment for Perinereis nigropunctata

XML Treatment for Perinereis obfuscata

XML Treatment for Perinereis singaporiensis

XML Treatment for Perinereis

XML Treatment for Platynereis

XML Treatment for Pseudonereis trimaculata

XML Treatment for Solomononereis marauensis

## Appendix 1

**Table A1.** Nereidid species data added and removed in the present study.

**No**.	**Species**	**Note**
**Addition**		
1	*Ceratonereis tentaculata*	Previously missing record
2	*Cheilonereis* sp.	New record
3	*Dendronereides cilacapensis*	New species
4	*Dendronereis javaensis*	New species
5	*Hediste* sp.	New record
6	*Neanthes bongcoi*	New record
7	Neanthes cf. gisserana	New species candidate
8	Neanthes cf. masalacensis	New species candidate
9	*Neanthes cricognatha*	Previously missing record
10	*Neanthes gisserana*	Previously missing record
11	Nereis cf. grayi	New record
12	*Nereis* sp_Ambon_NTMW19037	New species candidate and previously missing record
13	*Nereis trifasciata vandersandei*	Previously written as *Nereis vandersandei*
14	*Nicon pettiboneae*	New record
15	*Perinereis aibuhitensis*	Previously missing record
16	Platynereis cf. australis	Previously missing record
17	*Platynereis polyscalma*	Previously missing record
**Removal**		
1	*Gymnonereis fauveli*	Non-Indonesian species (the species belongs to Sri Lanka)
2	*Leonnates nierstraszi*	Non-Indonesian species (the species belongs to Philippines)
3	*Neanthes rubicunda*	From Nereis (Lycoris) gisserana; the correct species name is *Neanthes gisserana*
4	*Nereis vandersandi*	Unaccepted species name (accepted as *Nereis trifasciata vandersandei*)
5	*Perinereis camiguina*	*Taxon inquirendum*
6	*Perinereis cultrifera*	Non-Indonesian species
7	*Perinereis rumphii*	Unaccepted species name (accepted as *Perinereis nuntia*)
8	*Perinereis suluana*	Non-Indonesian species (the species belongs to Philippines)

## Additional information

### Artificial Intelligence (AI) use

The authors accept full responsibility for the content of the manuscript, including the disclosure of any use of AI.

No AI tools were used in the preparation of this manuscript.

### Author ORCIDs

J. Pamungkas https://orcid.org/0000-0003-1035-3056

C.J. Glasby https://orcid.org/0000-0002-9464-1938

G. Kerans https://orcid.org/0000-0001-5225-7981

L.P. Aji https://orcid.org/0000-0001-6017-3569

E. Tilic https://orcid.org/0000-0003-0463-322X

### Data availability

All of the data that support the findings of this study are available in the main text.
